# Endothelial PROX1 induces blood-brain barrier disruption in the central nervous system

**DOI:** 10.1172/jci.insight.187716

**Published:** 2026-01-09

**Authors:** Sara González-Hernández, Ryo Sato, Yuya Sato, Chang Liu, Wenling Li, Zulfeqhar A. Syed, Chengyu Liu, Sadhana Jackson, Yoshiaki Kubota, Yoh-suke Mukouyama

**Affiliations:** 1 Laboratory of Stem Cell and Neuro-Vascular Biology, Cell and Developmental Biology Center, National Heart, Lung, and Blood Institute,; 2 Electron Microscopy Core, National Heart, Lung, and Blood Institute,; 3 Transgenic Core, National Heart, Lung, and Blood Institute, and; 4 Developmental Therapeutics and Pharmacology Unit, Surgical Neurology Branch, National Institute of Neurological Disorders and Stroke, NIH, Bethesda, Maryland, USA.; 5 Department of Anatomy, Keio University School of Medicine, Shinjuku, Tokyo, Japan.

**Keywords:** Neuroscience, Vascular biology, Brain cancer, Endothelial cells, Tight junctions

## Abstract

The central nervous system (CNS) parenchyma has conventionally been believed to lack lymphatic vasculature, likely owing to a non-permissive microenvironment that hinders the formation and growth of lymphatic endothelial cells (LECs). Recent findings of ectopic expression of LEC markers including prospero homeobox 1 (PROX1), a master regulator of lymphatic differentiation, and the vascular permeability marker plasmalemma vesicle–associated protein (PLVAP) in certain glioblastomas (GBM) and brain arteriovenous malformations have prompted investigation into their roles in cerebrovascular malformations, tumor environments, and blood-brain barrier (BBB) abnormalities. To explore the relationship between ectopic LEC properties and BBB disruption, we used endothelial cell–specific *Prox1* overexpression mutants. When induced during embryonic stages of BBB formation, endothelial *Prox1* expression induces hybrid blood-lymphatic phenotypes in the developing CNS vasculature. This effect is not observed when *Prox1* is overexpressed during postnatal BBB maturation. Ectopic *Prox1* expression leads to significant vascular malformations and enhanced vascular leakage, resulting in BBB disruption when induced during both embryonic and postnatal stages. Mechanistically, PROX1 downregulates critical BBB-associated genes, including *β-catenin* and *claudin-5*, which are essential for BBB development and maintenance. These findings suggest that PROX1 compromises BBB integrity by negatively regulating BBB-associated gene expression and Wnt/β-catenin signaling.

## Abstract 2

Ectopic PROX1 expression in brain endothelial cells disrupts the blood-brain barrier (BBB), leading to vascular leakage by inhibiting key BBB-associated gene expression and Wnt/ß-catenin signaling.

## Graphical abstract

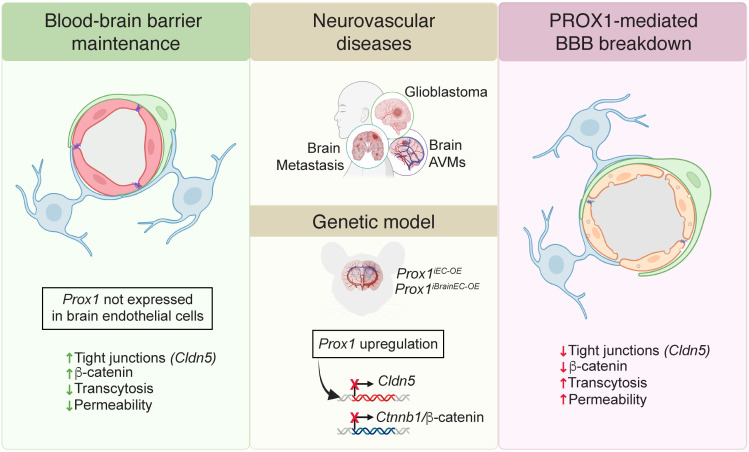

## Introduction

The central nervous system (CNS), comprising both the brain and spinal cord, develops a specialized vascular network characterized by the presence of specialized endothelial cells (ECs) that constitute the blood-brain barrier (BBB) and the absence of lymphatic vasculature within the parenchyma. This barrier serves as a formidable separation blockade, dividing the CNS from the peripheral blood circulation (1–5). The ECs constituting the BBB possess continuous intercellular tight junction (TJ) proteins, lack fenestrations, and display minimal transcytosis activity (1–5). Furthermore, it is plausible that the absence of classical, highly permeable lymphatic capillaries, which are composed of lymphatic ECs (LECs) with discontinuous button-like junctions, impedes the induction of an immune response to CNS-derived antigens. This establishes the CNS parenchyma as an organ with immune-privileged status (6–8). Blood and lymphatic vasculature are closely associated in non-CNS tissues; however, the link between BBB integrity and lymphatic avascularity in the CNS parenchyma remains poorly understood.

LEC specification relies on the action of the homeobox transcription factor prospero homeobox 1 (PROX1), which is necessary and sufficient to induce the LEC development program and repress the blood EC (BEC) development program in vitro and in vivo (9–15). Notably, LEC identity can be reprogrammed back into BEC identity by downregulation of the expression of PROX1 during embryonic, postnatal, or adult stages (13). While the CNS parenchyma is considered an organ devoid of lymphatic vasculature, recent studies demonstrate that PROX1^+^ lymphatic vasculature develops an extensive network in the dura mater of meninges under the skull (16–19), and PROX1^+^ non-lumenized mural LECs, also called brain LECs or fluorescent granule perithelial cells, develop in the surface of zebrafish brain and mammalian leptomeninges (20–24). In several pathological conditions, including glioblastoma (GBM) and brain arteriovenous malformations (AVMs), LEC markers including PROX1 are upregulated in ECs (25–27). Given that BBB integrity is often compromised in these GBM and AVMs, these findings suggest a potential link between ectopic LEC marker expression and BBB disruption. Under normal physiological conditions, suppression of LEC properties may be essential for the development and maintenance of BBB in the CNS parenchyma. However, in pathological conditions, the ectopic upregulation of LEC markers might contribute to BBB disruption, thereby promoting disease progression.

In this study, we first analyzed publicly available single-cell RNA sequencing (scRNA-Seq) data from human samples exhibiting impaired BBB integrity, including cases of GBM tumors (28–30), brain metastases (31), and AVMs (32). Our analysis reveals upregulation of lymphatic markers (*PROX1*, *LYVE1*, *FLT4*/*VEGFR3*) in the CNS vasculature across these diseases associated with BBB dysfunction, alongside increased levels of plasmalemma vesicle–associated protein (*PLVAP*), a protein commonly linked to endothelial permeability and BBB disruption. To explore the link between ectopic LEC marker expression and BBB disruption, we used a mouse model to express *Prox1* transgene, the master regulator of LEC development, in CNS ECs during BBB formation or maintenance. EC-specific overexpression of *Prox1* in mice results in notable alterations in the morphology and barrier function of the CNS vasculature. Interestingly, endothelial *Prox1* expression induces a hybrid blood-lymphatic phenotype, characterized by the expression of both BEC markers and a subset of LEC markers, in the developing CNS vasculature when induced during primitive BBB formation at embryonic stages. However, such a hybrid blood-lymphatic phenotype is not observed when the *Prox1* expression is induced during the BBB maturation at postnatal stages. Endothelial *Prox1* expression promotes enhanced vascular leakage and BBB disruption when induced during both embryonic and postnatal stages. Importantly, using a brain EC–specific *Slco1c1-Cre^ERT2^* driver (33), we demonstrate that *Prox1* expression within CNS ECs alone is sufficient to disrupt BBB integrity, confirming a cell-autonomous role for *Prox1* in the brain vasculature. This vascular leakage is attributed to the downregulation of TJ proteins and the upregulation of transcytosis, underscoring the inhibitory effects of PROX1 on BBB development and maintenance. At the molecular level, PROX1 reduces the mRNA expression of BBB-associated genes, including *Ctnnb1* (β-catenin), which is a critical signaling component for BBB development and maintenance. These studies suggest the potential clinical implications of regulating *Prox1* in the CNS vasculature.

## Results

### LEC markers are upregulated in ECs within brain tumors and vascular malformations.

**Figure 1:**
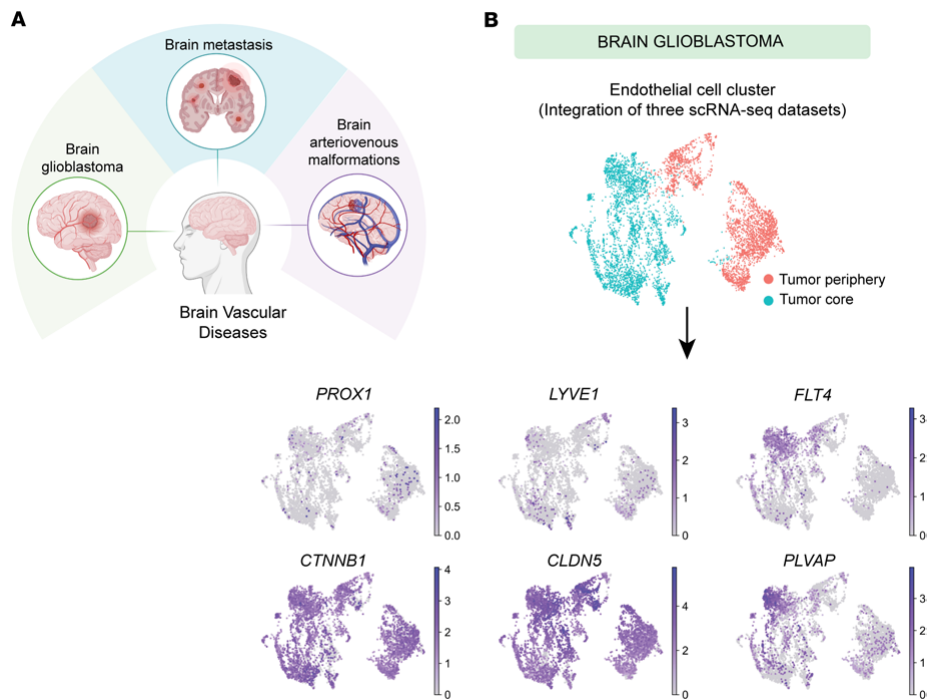
LEC markers and the vascular permeability marker *PLVAP* are upregulated in ECs from glioblastoma. (**A**) Schematic representation of human brain vascular diseases analyzed using publicly available scRNA-Seq datasets: glioblastoma (GBM) (28–30), brain metastasis (31), and arteriovenous malformations (AVMs) (32). Created in BioRender (Gonzalez S, 2025, https://BioRender.com/kdo2upo). (**B**) UMAP plots of scRNA-Seq data integrated from three GBM datasets display EC clusters expressing LEC markers (*PROX1*, *LYVE1*, *FLT4*) and the vascular permeability marker *PLVAP*, along with brain EC markers (*CTNNB1*, *CLDN5*).

**Figure 2:**
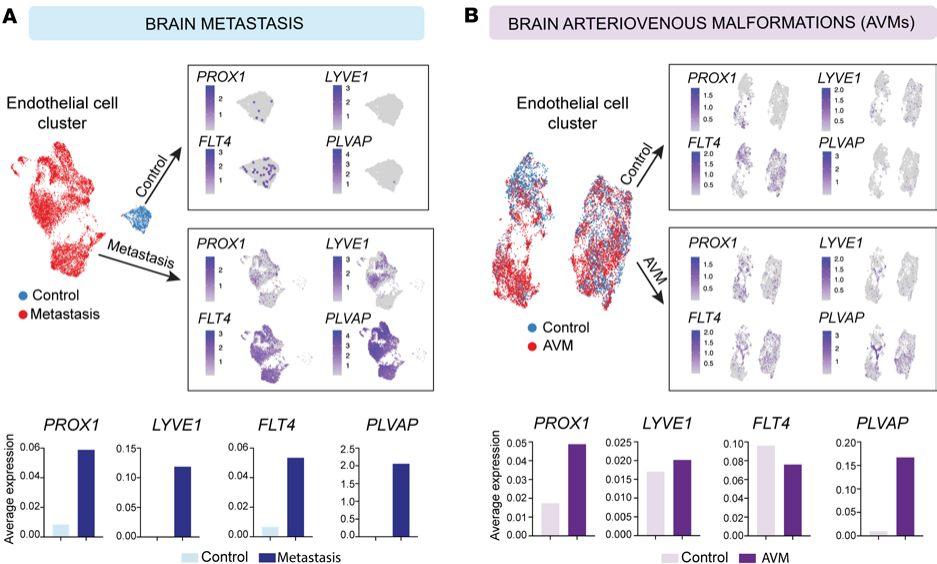
Upregulation of LEC markers and the vascular permeability marker *PLVAP* in ECs from brain metastasis and AVMs. (**A**) UMAP plots and average gene expression charts of scRNA-Seq data of control and metastatic brain tumor datasets display EC clusters expressing LEC markers, including *PROX1*, *LYVE1*, and *FLT4*, along with *PLVAP*. (**B**) UMAP plots and average gene expression charts of scRNA-Seq data of control and AVM datasets display EC clusters expressing LEC markers, including *PROX1*, *LYVE1*, and *FLT4*, along with *PLVAP*.

We analyzed publicly available scRNA-Seq datasets from human GBM (28–30), brain metastases (31), and AVMs (32) to assess the expression of LEC markers in ECs (Figure 1A). After extracting ECs from 3 GBM datasets and integrating them (Supplemental Figure 1, A and B; supplemental material available online with this article; https://doi.org/10.1172/jci.insight.187716DS1), we observed *PROX1* expression in ECs within the tumors, accompanying other LEC markers (*LYVE1* and *FLT4*) (Figure 1B). Notably, *PROX1* expression appeared to be scattered rather than confined to a single EC cluster (Figure 1B and Supplemental Figure 1B), which likely reflects tumor heterogeneity and integration across 3 independent studies. In contrast, the brain metastasis and AVM datasets each comprised disease (red) and control (blue) conditions, allowing for direct comparisons between these states (Figure 2, A and B, and Supplemental Figure 1, C and D). Examination of LEC genes revealed a pronounced increase in *PROX1* under disease conditions in both datasets. Additionally, *PLVAP*, which is commonly associated with endothelial permeability and BBB disruption (34–36), was increased across all 3 disease conditions (Figure 1B and Figure 2, A and B). These data suggest that LEC-associated transcripts are upregulated in CNS ECs in pathology and may be linked to vascular permeability and BBB disruption.

To investigate a potential link between ectopic LEC marker upregulation in the CNS parenchyma and BBB disruption, we turned to a mouse model to manipulate *Prox1* expression in the brain vasculature during embryonic BBB formation or postnatal BBB maturation. Based on the scRNA-Seq analysis indicating the presence of rare PROX1^+^ ECs in control human brain samples, we began by examining *Prox1* expression in the mouse brain and spinal cord using the *Prox1-Gfp BAC* transgenic reporter (37), which labels PROX1-expressing cells with the green fluorescent protein (GFP). Since *Prox1* is also expressed in neural progenitors (38), we defined PROX1-expressing ECs as those cells that colocalize GFP with the pan-EC marker PECAM1 and the EC nuclear marker ERG. We also confirmed the specificity of the GFP labeling using an anti-PROX1 antibody.

At embryonic day 13.5 (E13.5), section immunostaining showed that ERG^+^ ECs in the brain and spinal cord did not colocalize with PROX1 or GFP, whereas many neural progenitors were ERG^–^PROX1^+^GFP^+^ (Supplemental Figure 2, A–C; arrows indicate ERG^+^ EC nuclei). Similarly, spinal cord parenchyma ERG^+^ ECs lacked PROX1 and GFP (Supplemental Figure 2, D and E, arrows). At E15.5, Prox1-GFP remained absent from PECAM1^+^ERG^+^ brain ECs (Supplemental Figure 2F; arrows indicate ERG^+^PECAM1^+^ ECs), and this persisted postnatally (Supplemental Figure 2G).

Importantly, costaining with PECAM1 and LYVE1 confirmed the absence of classical lymphatic vessels (PECAM1^+^LYVE1^+^Prox1-GFP^+^) in the brain parenchyma at postnatal day 3 (P3). Only LYVE1^+^ macrophages (PECAM1^–^LYVE1^+^Prox1-GFP^–^) were found in perivascular regions (Supplemental Figure 2, G and H, yellow arrowheads, and Supplemental Figure 3, A–C). In contrast, PECAM1^+^LYVE1^+^Prox1-GFP^+^ lymphatic vessels were observed in meningeal layers and head skin vasculature (Supplemental Figure 2, I and J, arrowheads, and Supplemental Figure 3, D–H, arrows). Combined, this time course analysis reaffirms the dearth of lymphatic vasculature within the CNS parenchyma and the lack of the lymphatic master regulator PROX1 in CNS ECs under physiological conditions.

### Endothelial Prox1 expression leads to severe vascular abnormalities in the developing CNS vasculature.

**Figure 3:**
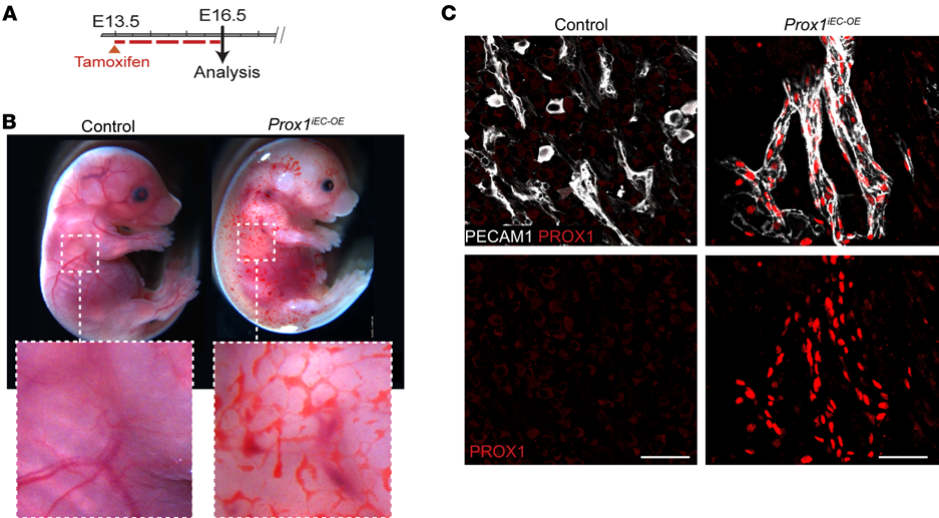
Endothelial *Prox1* overexpression during embryonic stages. (**A**) Diagram depicting EC-specific induction of *Prox1* expression at E13.5 and analysis of embryos at E16.5. (**B**) Gross appearance of *Prox1^iEC-OE^* mutant and control embryos. The mutant embryos display blood-filled lymphatic vasculature in the skin. The regions in dashed boxes are magnified in the inset panels. (**C**) Section immunostaining of E16.5 *Prox1^iEC-OE^* mutant and their control littermate brains with antibodies against PROX1 (red) and PECAM1 (gray). *Prox1* expression is induced in the brain vasculature (gray) of E16.5 *Prox1^iEC-OE^* mutants compared with their control littermates. Scale bars: 50 μm.

To address the relationship between PROX1 and BBB development/maintenance in a non-disease context, we generated conditional *Prox1* overexpression mice harboring a *loxP-STOP-loxP-Prox1* cassette in the *Rosa26* locus (*R26-LSL-Prox1*) (39), allowing time- and cell type–specific induction (Supplemental Figure 4A). We crossed these to the EC-specific *Cdh5-BAC-Cre^ERT2^* driver (40) to induce the *Prox1* transgene in ECs. Since the primitive BBB becomes functional around E15.5 (41), we opted to induce the *Prox1* transgene in *R26-LSL-Prox1* embryos (hereafter referred to as *Prox1^iEC-OE^*) through tamoxifen administration at E13.5 and examine the resulting impact on brain vasculature development and BBB integrity at E16.5 (Figure 3A). It is important to note that *Cdh5-Cre^ERT2^* mice are widely used as an EC-specific *Cre^ERT2^* driver, but the *Cdh5* promoter/enhancer is preferentially, but not exclusively, active in vascular ECs. Therefore, we carefully analyzed the *Prox1* transgene expression in *Prox1^iEC-OE^* mice.

**Figure 4:**
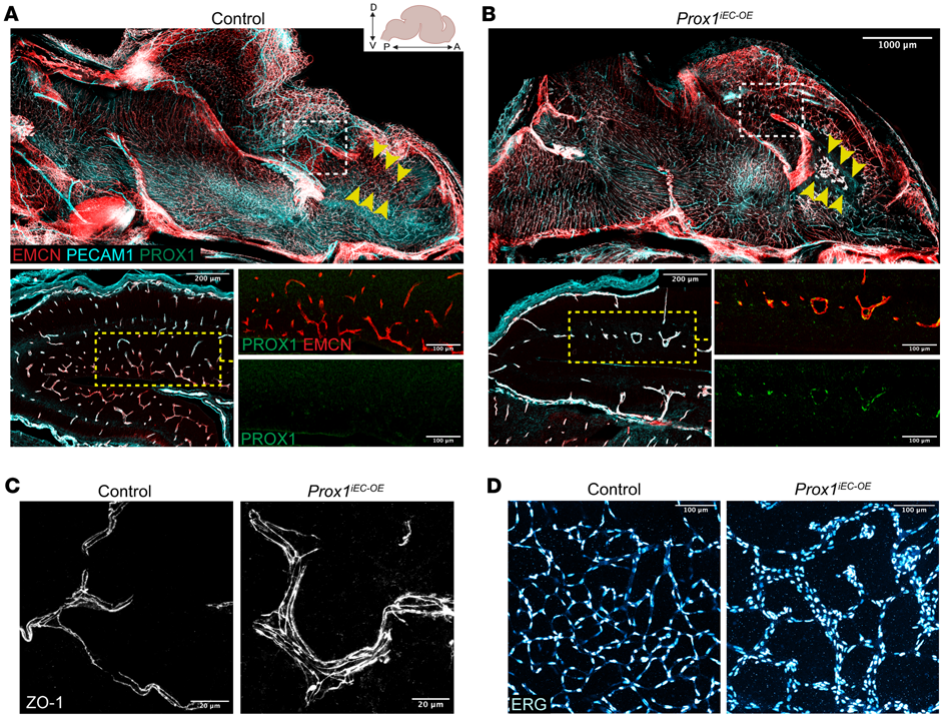
Endothelial *Prox1* overexpression disrupts CNS vascular development. (**A** and **B**) A sagittal view of whole-mount immunostaining of E16.5 *Prox1^iEC-OE^* mutant (**B**) and their control littermate brains (**A**) labeled with EMCN (red), PECAM1 (cyan) and PROX1 (green). Yellow arrowheads indicate cortical vasculature. White boxed regions in **A** and **B** are shown at higher magnification below. *Prox1^iEC-OE^* mutant brains exhibited PROX1^+^EMCN^+^ enlarged capillaries (yellow boxed inset in **B**) in comparison to their control littermates (yellow boxed inset in **A**). Created in BioRender (Gonzalez S, 2025, https://BioRender.com/kdo2upo). (**C** and **D**) Section immunostaining of E16.5 *Prox1^iEC-OE^* mutant and their control littermate brains with ZO-1 (**C**, gray) and ERG (**D**, hot cyan). *Prox1^iEC-OE^* mutants exhibited enlarged capillaries with an increased number of ECs in comparison to their control littermates. *Prox1^iEC-OE^* mutants exhibited enlarged capillaries with an increased number of ECs in comparison to their control littermates. Scale bars: 1,000 μm (**A** and **B**, top), 200 μm (**A** and **B**, bottom left), 100 μm (**A** and **B**, bottom right, and **D**), 20 μm (**C**).

*Prox1^iEC-OE^* mutant embryos exhibited pronounced edema, hemorrhage, and blood-filled lymphatics in skin (Figure 3B and Supplemental Figure 4B) and embryonic lethality within 72 hours of induction. We validated the efficient induction of the *Prox1* transgene in PECAM1^+^ brain ECs of *Prox1^iEC-OE^* mutant embryos, whereas control littermates lacked PROX1 in ECs (Figure 3C). Sagittal overviews highlight notable disparities in the brain vasculature between *Prox1^iEC-OE^* mutant embryos and their control littermates, notably in the cerebral cortex region where abnormal enlarged vessels were present, while capillary density was reduced in the mutants (Figure 4, A and B, yellow arrowheads, and Supplemental Figure 4, C–H). Immunostaining with antibodies against the adherent junction marker ZO-1 (TJP1) and ERG revealed the formation of thick capillaries due to an augmented number of ECs in *Prox1^iEC-OE^* mutant embryos, as compared with their control littermates (Figure 4, C and D).

**Figure 5:**
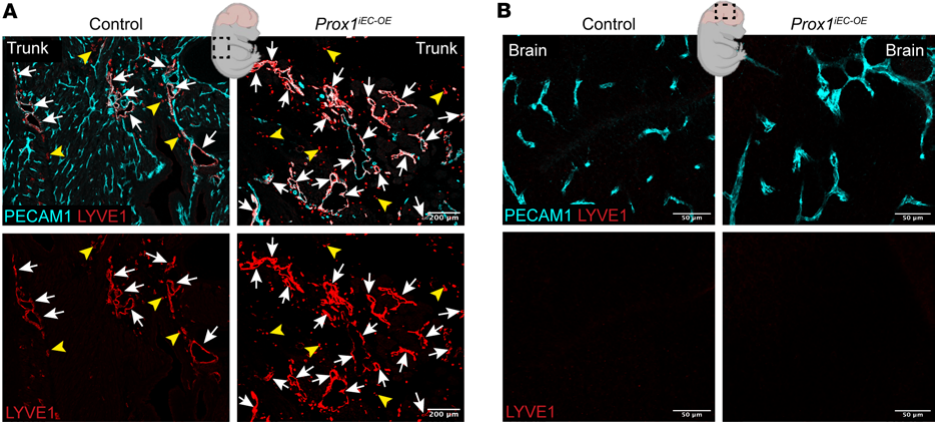
Endothelial *Prox1* does not induce conventional lymphatic vessels in developing CNS vasculature. (**A** and **B**) Section immunostaining of trunk (**A**) and brain (**B**) from E16.5 *Prox1^iEC-OE^* and control embryos stained for PECAM1 (cyan) and LYVE1 (red). Arrows indicate PECAM1^+^LYVE1^+^ lymphatic vessels; yellow arrowheads mark PECAM1^–^LYVE1^+^ macrophages. *Prox1^iEC-OE^* mutants exhibited enhanced lymphatic differentiation to form PECAM1^+^LYVE1^+^ lymphatic vasculature in the trunk in comparison with their control littermates. In contrast, both *Prox1^iEC-OE^* mutants and their control littermates did not exhibit conventional PECAM1^+^LYVE1^+^ lymphatic vasculature in the brain. Scale bars: 200 μm (**A**), 50 μm (**B**). Created in BioRender (Gonzalez S, 2025, https://BioRender.com/kdo2upo).

We next investigated whether *Prox1* expression induces an LEC fate in the CNS vasculature of *Prox1^iEC-OE^* embryos. We first examined the expression of the classical LEC marker LYVE1 in the vasculature of *Prox1^iEC-OE^* mutants and their control littermates. We observed a substantial increase in PECAM1^+^LYVE1^+^ lymphatic vessels in trunk vasculature of mutants versus control (Figure 5A, arrows), but not LYVE1^+^ ECs in the brain of either genotype (Figure 5B). Quantitative validation of these findings was achieved through flow cytometry/fluorescence-activated cell sorting (FACS) analysis (Supplemental Figure 5, A and B): PECAM1^+^LYVE1^+^ LECs were undetectable among brain ECs in both groups (constituting 0% of brain ECs), whereas mutant skin showed increased LYVE1^+^PECAM1^+^ LECs (from 5% to 30% of skin ECs) and decreased LYVE1^–^PECAM1^+^ BECs (from 95% to 70% of skin ECs). These data suggest that, consistent with the established propensity of PROX1 function to evoke lymphatic differentiation in the developing vasculature, endothelial *Prox1* expression induces the differentiation of BECs into LECs in the skin vasculature. In contrast, in the brain vasculature, *Prox1* does not induce conventional LECs. While *Prox1* induces notable remodeling in the brain parenchymal vasculature, characterized by the rapid development of enlarged vessels and thicker capillaries, particularly in the cerebral cortex region, it appears that *Prox1* expression alone is insufficient to induce conventional LECs expressing the classical LEC markers such as LYVE1 (Figure 5B) and podoplanin (PDPL, data not shown).

Notably, given our use of the EC-specific *Cdh5-BAC-Cre^ERT2^* driver mice to induce the *Prox1* transgene in ECs, we observed abnormalities in the lymphatic vasculature in peripheral tissues. For instance, whole-mount immunostaining of limb skin and heart ventricles revealed aberrant branching of lymphatic vessels in *Prox1^iEC-OE^* mutant embryos (Supplemental Figure 5, C–E). As previously described (42), LYVE1^+^PECAM1^+^ cardiac lymphatic vessels extended inferior on both the ventral and dorsal surfaces of the heart ventricle in the control littermates (Supplemental Figure 5D, arrows). Notably, some of these lymphatic vessels branched closely to EMCN^+^PECAM1^+^ large-diameter coronary veins on the dorsal surface of the heart ventricle. In contrast, the ventral surface of the mutant heart ventricle exhibited blood-filled lymphatic vasculature, while the dorsal surface showed abnormal lymphatic structures (Supplemental Figure 5E). Additionally, the mutants exhibited underdeveloped coronary vasculature, characterized by the absence of large-diameter coronary arteries (Supplemental Figure 5, D and E, PECAM1^+^, white arrowheads) and veins (Supplemental Figure 5, D and E, EMCN^+^, yellow arrowheads). These findings suggest that endothelial *Prox1* expression leads to abnormal coronary and cardiac lymphatic vasculature in the developing heart ventricles.

### Endothelial Prox1 expression induces a hybrid blood-lymphatic phenotype in the developing CNS vasculature.

**Figure 6:**
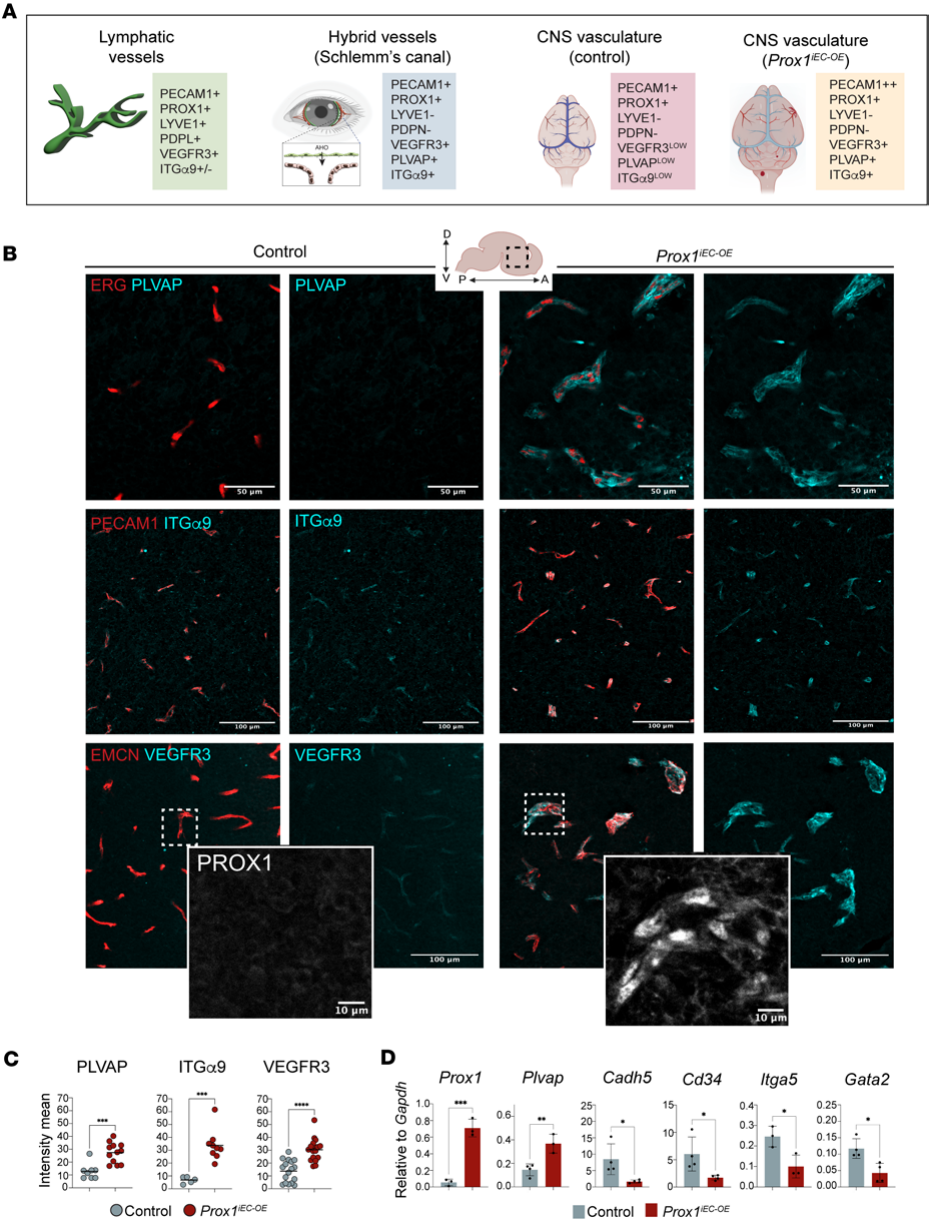
Endothelial *Prox1* induces a hybrid blood-lymphatic phenotype in developing CNS vasculature. (**A**) Schematic illustrations indicating EC markers expressed in conventional lymphatic vessels, hybrid vessels in the Schlemm’s canal, and CNS vessels in controls and *Prox1^iEC-OE^* mutants. (**B**) Section immunostaining of E16.5 *Prox1^iEC-OE^* mutant and their control littermate brains with PLVAP (cyan), ITGα9 (cyan), and VEGFR3 (cyan), together with ERG (red), PECAM1 (red), and EMCN (red), respectively. The sections labeled with VEGFR3 (cyan) and EMCN (red) are additionally stained with PROX1 (gray in the magnified images). (**C**) Quantifications of the mean fluorescence intensity for PLVAP, ITGα9, and VEGFR3 in the brain vasculature using Imaris software (Oxford Instruments). Each dot corresponds to random fields of view from at least 3 different control and mutant embryos. Data shown as mean ± SEM. (**D**) Relative mRNA expression levels of *Prox1* and *Plvap* together with BEC markers such as *Cadh5*, *Cd34*, *Itga5*, and *Gata2* in FACS-isolated brain ECs from E16.5 *Prox1^iEC-OE^* mutant and their control littermate brains. Bar graphs show mean normalized expression ± SEM; *n* = 3–4 biological samples obtained from FACS-isolated brain ECs from individual experiments. Mean ± SEM, unpaired *t* test. *P < 0.05, **P < 0.001, ***P < 0.0005, ****P < 0.0001. Scale bars: 50 μm (top panels), 100 μm (middle and bottom panels), 10 μm (inset panels). Created in BioRender (Gonzalez S, 2025, https://BioRender.com/kdo2upo).

In light of the recent discovery of Schlemm’s canal in the eye — a specialized ring-shaped vasculature at the periphery of the cornea with ECs that have BEC and LEC characteristics, including the expression of BEC markers and a subset of LEC makers (43–46) — we proceeded to examine whether *Prox1* expression induces a similar hybrid phenotype in the brain vasculature. Schlemm’s canal ECs manifest the expression of BEC markers including PECAM1, EMCN, CD34, CDH5 (VE-cadherin), and TIE2, together with LEC markers PROX1, VEGFR3, and ITGα9. The classical LEC markers LYVE1 and PDPL are absent in Schlemm’s canal ECs (Figure 6A). Additionally, PLVAP, a component of endothelial fenestrae that regulates basal permeability (34–36), is highly expressed in Schlemm’s canal ECs (Figure 6A).

In the brain vasculature of control littermates, the expression of PLVAP, VEGFR3, and ITGα9 was scarcely detectable in ECs (Figure 6B; overlap with the pan-EC markers ERG and PECAM1, or the pan-capillary EC marker EMCN). In contrast, in the brain vasculature of *Prox1^iEC-OE^* mutant embryos, these markers were substantially upregulated (Figure 6B and quantification in Figure 6C). At the transcript level, brain ECs isolated through FACS from *Prox1^iEC-OE^* embryos demonstrated increased expression of *Plvap* compared with controls (Figure 6D). Although the expression of BEC markers such as *Cdh5*, *Cd34*, *Itga5*, and *Gata2* was partially reduced in *Prox1^iEC-OE^* mutants (Figure 6D), it is evident that endothelial *Prox1* expression does not completely reprogram BECs to LECs in the brain vasculature. Taken together, this evidence shows that *Prox1* induces a hybrid blood-lymphatic phenotype in the brain vasculature, reminiscent of Schlemm’s canal ECs in the eyes, with the expression of BEC (PECAM1^+^PLVAP^+^) and LEC (PROX1^+^VEGFR3^+^ITGα9^+^) markers.

### Endothelial expression of Prox1 disrupts primitive BBB formation in the developing CNS vasculature.

CNS ECs express the TJ protein claudin-5 (CLDN5) as a hallmark of BBB integrity. In contrast, PLVAP, which is associated with high-permeability vasculature, is normally absent (5, 47). In compromised BBB regions, CLDN5 decreases and PLVAP is induced (5, 48). We therefore investigated whether the acquisition of such a hybrid blood-lymphatic phenotype in the CNS vasculature of *Prox1^iEC-OE^* embryos might affect the development and integrity of the BBB.

**Figure 7:**
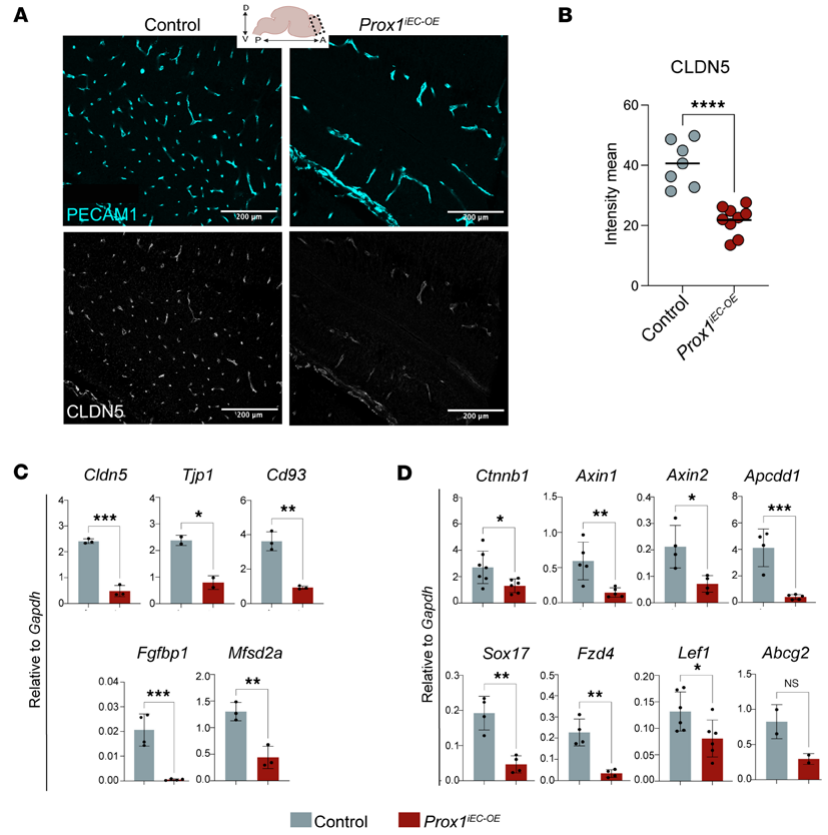
Endothelial *Prox1* alters BBB-associated gene expression. (**A**) Section immunostaining of E16.5 *Prox1^iEC-OE^* mutant and their control littermate brains with PECAM1 (cyan) and CLDN5 (gray). The expression of CLDN5 is downregulated in the brain vasculature of *Prox1^iEC-OE^* mutants in comparison with their control littermates. Created in BioRender (Gonzalez S, 2025, https://BioRender.com/kdo2upo). (**B**) Quantification of the mean fluorescence intensity for CLDN5 in the brain vasculature using Imaris software. Each dot corresponds to random fields of view from at least 3 different control and mutant embryos. Mean ± SEM, unpaired *t* test. ****P < 0.0001. (**C** and **D**) Relative mRNA expression levels of BBB-related genes such as *Cldn5*, *Tjp1*, *Cd93*, *Fgfbp1*, and *Mfsd2a* in **C**, and *Ctnnb1* and its target genes such *Axin1*, *Axin2*, *Apcdd1*, *Sox17*, *Fzd4*, *Lef1*, and *Abcg2* in **D**, in FACS-isolated brain ECs from E16.5 *Prox1^iEC-OE^* mutant and their control littermate embryos. *n* = 3–5 obtained from 4 individual experiments. Mean ± SEM, unpaired *t* test. *P < 0.05, **P < 0.001 ***P < 0.0005. Scale bars: 200 μm.

Section immunostaining for CLDN5 and the pan-EC marker PECAM1 clearly demonstrated a reduction in CLDN5 expression in the brain vasculature of *Prox1^iEC-OE^* embryos compared with their control littermates (Figure 7A and quantification in Figure 7B). Similar results were obtained with whole-mount immunostaining of the brain vasculature labeled with EMCN and CLDN5 markers (Supplemental Figure 6, A–C). This reduction indicates impaired TJ assembly among cerebral ECs, suggesting a defect in barrier integrity. Given that *Cdh5-Cre^ERT2^* is expressed in both BECs and LECs, our model induces *Prox1* overexpression in both endothelial populations. However, quantitative reverse transcription PCR analysis of FACS-isolated cells revealed that *Cldn5* expression in skin LECs was not significantly affected by *Prox1* overexpression (Supplemental Figure 6D), suggesting that *Cldn5* regulation remains largely unaltered in LECs under these conditions.

**Figure 8:**
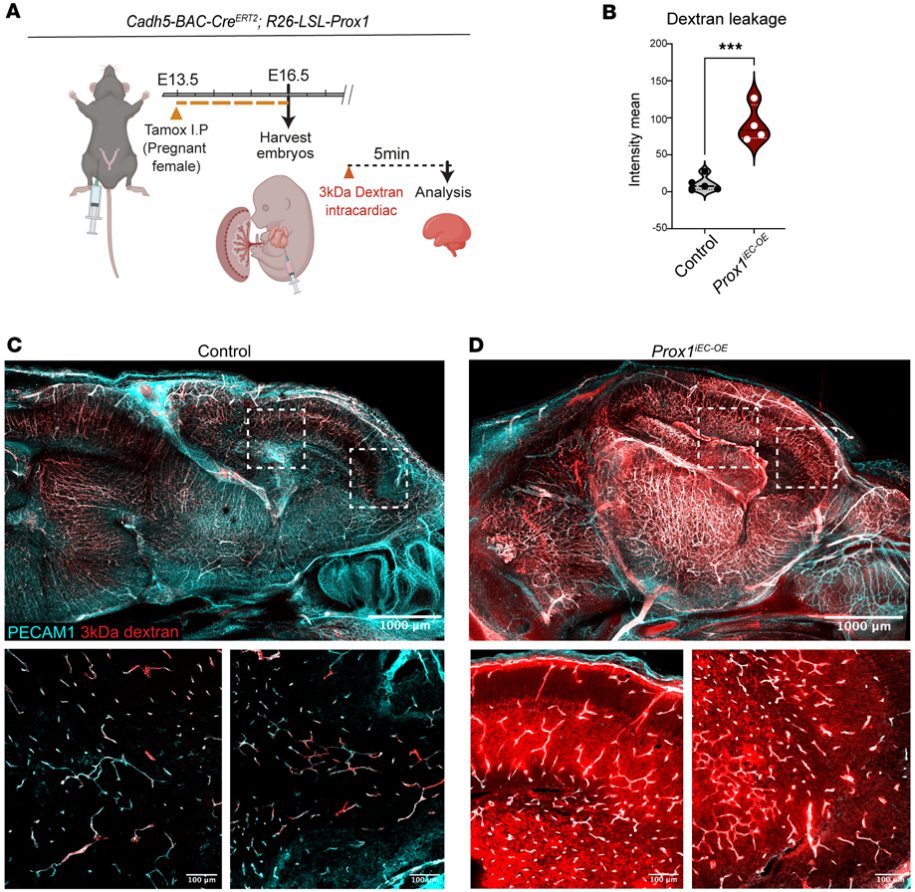
Endothelial *Prox1* expression disrupts the primitive BBB formation during CNS development. (**A**) Diagram depicting EC-specific *Prox1* induction at E13.5 and vascular permeability assay at E16.5. Created in BioRender (Gonzalez S, 2025, https://BioRender.com/kdo2upo). (**B**) Quantification of 3 kDa dextran extravasation outside of the brain vasculature in control (*n* = 5 individual brains, showing the average of 4 different fields of view) and mutant brains (*n* = 4 individual brains, showing the average of 4 different fields of view). Mean ± SEM, unpaired *t* test. ***P < 0.0005. (**C** and **D**) A sagittal view of whole-mount imaging of E16.5 *Prox1^iEC-OE^* mutant (**D**) and their control littermate (**C**) brains with 3 kDa dextran (red) and PECAM1 (cyan). Boxed regions in **C** and **D** are shown at higher magnification below. Scale bars: 1,000 μm (**C** and **D**, top),100 μm (**C** and **D**, bottom).

Consistent with barrier compromise, we observed TER119^+^ blood cell extravasation in *Prox1^iEC-OE^* brains (Supplemental Figure 6, E–G, arrows). To further address the BBB function, we performed a tracer leakage assay at the stage when the primitive BBB becomes functional (41). We harvested E16.5 embryos and performed an intracardial injection of a 3 kDa fluorescent tracer, dextran Texas red (Figure 8A). Whole-brain imaging and subsequent immunostaining of sagittal brain samples revealed extensive BBB leakage in *Prox1^iEC-OE^* mutants compared with control embryos (Figure 8, C and D, quantification in Figure 8B, and Supplemental Figure 6, H–J): In the control embryos, the injected dextran tracer remained entirely within PECAM1^+^ vasculature (Figure 8, C and D, and Supplemental Video 1). However, severe BBB leakage was observed in the mutant brains, particularly within the cerebral cortex (Figure 8, C and D, and Supplemental Video 2). These findings indicate that endothelial *Prox1* expression disrupts primitive BBB formation in the developing CNS vasculature.

We next assessed the mRNA expression of BBB markers in FACS-isolated brain ECs. We observed a decrease in the expression of TJ markers *Cldn5* and *Tjp1* in *Prox1^iEC-OE^* mutants compared with their control brain ECs (Figure 7C). We also observed a decrease in the expression of recently identified BBB-related genes, such as *Cd93* (49) and *Fgfbp1* (50), in the mutant embryos compared with their control littermates (Figure 7C). Additionally, we found a reduction in the expression of the lipid transporter *Mfsd2a*, which plays an essential role in limiting caveolin-dependent transcytosis in BBB ECs (41, 51–53), in *Prox1^iEC-OE^* brain ECs. Furthermore, the expression of *Pten*, which serves as an upstream regulator of the Mfsd2a-transcytosis axis (53), was also downregulated in mutant CNS vasculature (Supplemental Figure 6K). This finding suggests a potential upregulation of transcytosis in addition to an impaired TJ upon *Prox1* overexpression. Given that Wnt/β-catenin signaling is known to regulate many BBB genes, including *Cldn5*, *Plvap*, and *Mfsd2a* (1, 4, 5), we observed a decrease in the expression of *Ctnnb1* as well as several effector and target genes associated with Wnt/β-catenin signaling in the mutant embryos compared with their control littermates (Figure 7D and Supplemental Figure 6K). These results indicate that the endothelial *Prox1* expression leads to a significant downregulation of Wnt/β-catenin signaling in the developing CNS vasculature.

Pericyte-EC association is essential for the formation of a functionally effective BBB (54, 55). Thus, barrier defects in *Prox1^iEC-OE^* embryos could be due to altered pericyte coverage of capillaries. However, immunostaining for pericyte markers NG2 and PDGFRβ, in combination with PECAM1, revealed pericyte coverage of enlarged capillaries in the brain vasculature of *Prox1^iEC-OE^* mutants (Supplemental Figure 6, L–O, arrows). Indeed, FACS analysis revealed a comparable number of CD140b(PDGFRβ)^+^CD31(PECAM1)^–^ pericytes in both groups, exhibiting a similar maximal fluorescence intensity (MFI) (Supplemental Figure 6, P and Q). Notably, NG2 also labeled oligodendrocyte-lineage cells (NG2^+^PDGFRβ^–^), and we observed increased association with capillaries (Supplemental Figure 6, L–O, yellow arrowheads) in *Prox1^iEC-OE^* brains compared with controls. Given that previous studies have reported the expression of Wnt7a/b ligands for canonical Wnt/β-catenin signaling (56–58) by oligodendrocytes, in addition to astroglia and neurons, these findings suggest a potential role in repairing BBB disruption.

### Postnatal induction of Prox1 leads to BBB breakdown.

**Figure 9:**
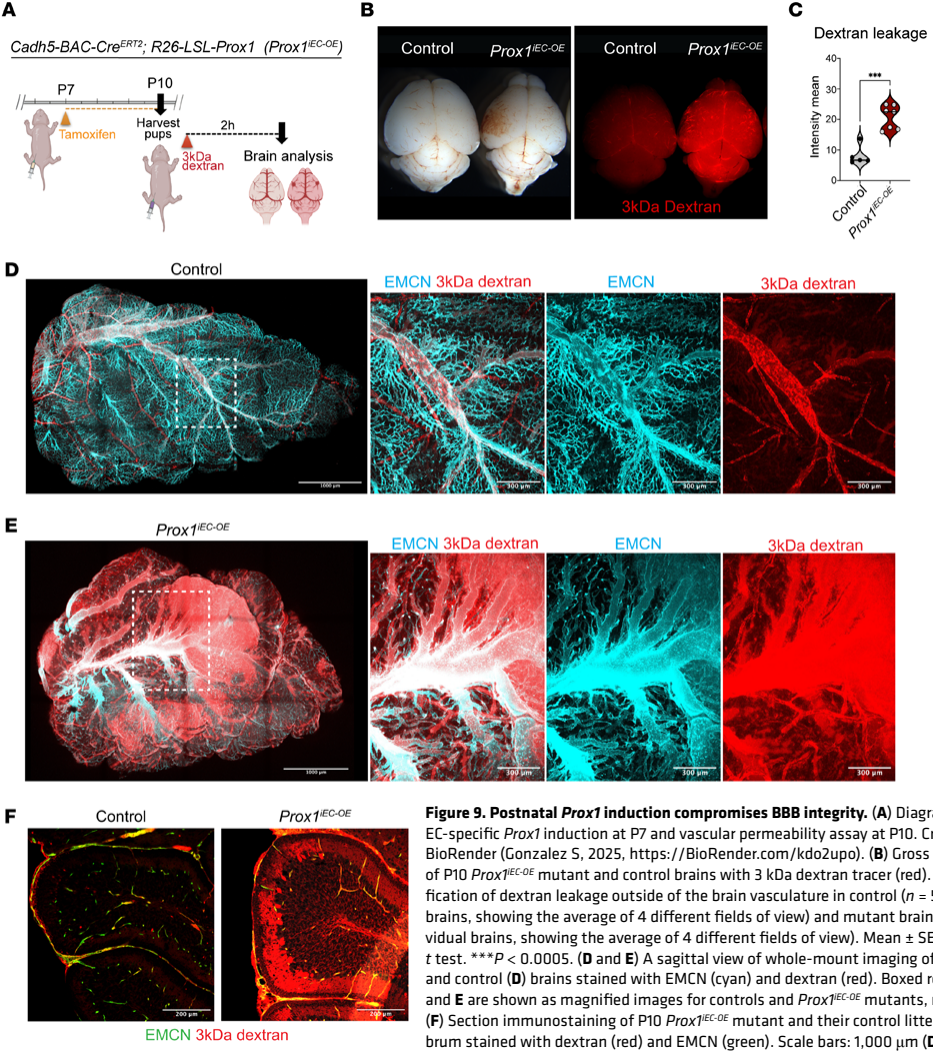
Postnatal *Prox1* induction compromises BBB integrity. (**A**) Diagram depicting EC-specific *Prox1* induction at P7 and vascular permeability assay at P10. Created in BioRender (Gonzalez S, 2025, https://BioRender.com/kdo2upo). (**B**) Gross appearance of P10 *Prox1^iEC-OE^* mutant and control brains with 3 kDa dextran tracer (red). (**C**) Quantification of dextran leakage outside of the brain vasculature in control (*n* = 5 individual brains, showing the average of 4 different fields of view) and mutant brains (*n* = 7 individual brains, showing the average of 4 different fields of view). Mean ± SEM, unpaired *t* test. ***P < 0.0005. (**D** and **E**) A sagittal view of whole-mount imaging of *Prox1^iEC-OE^* (**E**) and control (**D**) brains stained with EMCN (cyan) and dextran (red). Boxed regions in **D** and **E** are shown as magnified images for controls and *Prox1^iEC-OE^* mutants, respectively. (**F**) Section immunostaining of P10 *Prox1^iEC-OE^* mutant and their control littermate cerebrum stained with dextran (red) and EMCN (green). Scale bars: 1,000 μm (**D** and **E**), 300 μm (**D** and **E**, 3 right images), and 200 μm (**F**).

The observation that endothelial *Prox1* expression during primitive BBB formation led to barrier disruption prompted us to investigate whether PROX1 itself compromises the mature BBB, even in the absence of LEC differentiation in the CNS parenchyma. To address this question, we induced the *Prox1* transgene at P7 and assessed BBB function at P10. Tracer leakage assays were performed with intraperitoneal injection of a 3 kDa dextran Texas red or a 1 kDa Alexa Fluor 555–cadaverine (Figure 9A) in control and *Prox1^iEC-OE^* mutants. Bright-field whole-brain images showed enlarged vessels and hemorrhages in the surface of *Prox1^iEC-OE^* brains compared with control littermates (Figure 9B). Whole-mount immunostaining and tissue clearing of sagittal brain samples with antibodies against the EC marker PECAM1 or EMCN revealed extensive BBB leakage in *Prox1^iEC-OE^* brains (Figure 9, D and E, and quantification in Figure 9C). Severe BBB leakage was observed within the mutant vasculature with extensive vascular malformations and vessel enlargement in the surface of the brain (Figure 9, D and E, and Supplemental Figure 7A). Subsequent section immunostaining of the cerebellum clearly demonstrated that the dextran tracer leaked out of vessels in the mutant mice (Figure 9F). We also observed similar leakage using the 1 kDa cadaverine tracer (Supplemental Figure 7B and quantification in Supplemental Figure 7C).

We next investigated whether *Prox1* expression impacts the capillary network and BBB integrity. While the control brain exhibited a dense capillary network, the mutant brain displayed abnormally enlarged vasculature, characterized by reduced vascular density and larger-caliber vessels (Supplemental Figure 7A). However, we did not observe any significant change in the mRNA expression of BEC markers such as *Cdh5*, *Cd34*, *Itga5*, and *Gata2* between *Prox1^iEC-OE^* mutants and their control littermates (Supplemental Figure 7E). Moreover, we found no evidence of hybrid blood-lymphatic phenotype in postnatal *Prox1^iEC-OE^* mutants, as LEC markers VEGFR3 and ITGα9 were not upregulated (Supplemental Figure 7, G and H). These findings suggest that *Prox1* does not reprogram postnatal brain vasculature into a hybrid state.

**Figure 10:**
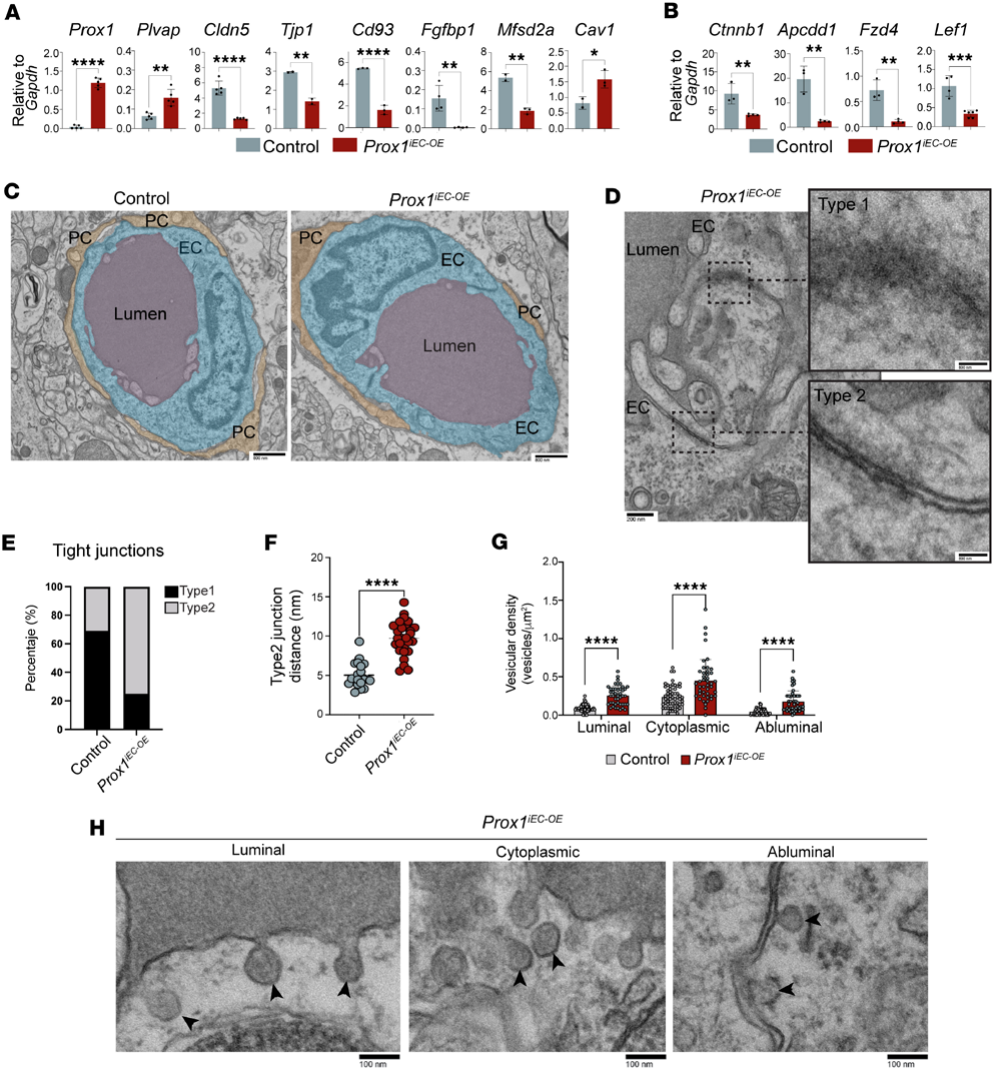
Postnatal *Prox1* induction disrupts TJs and increases transcytosis. (**A** and **B**) Relative mRNA expression levels of *Prox1* and *Plvap* together with BBB-related genes such as *Cldn5*, *Tjp1*, *Cd93*, *Fgfbp1*, *Mfsd2a*, and *Cav1* in **A**, and *Ctnnb1* and its target genes such *Apcdd1*, *Fzd4*, and *Lef1* in **B**, in FACS-isolated brain ECs from P10 *Prox1^iEC-OE^* and controls. *n* = 3–4 biological samples obtained from FACS-isolated brain ECs from individual experiments. Mean ± SEM, unpaired *t* test. (**C**–**F**) Transmission electron microscopy (TEM) of brain capillaries from control (*n* = 5) and *Prox1^iEC-OE^* mutant brains (*n* = 6). (**C**) Representative images of brain capillaries with color mask applied to identify the lumen of the vessels, ECs, and pericytes (PC). (**D**) Representative TEM image of *Prox1^iEC-OE^* mutant brain showing type 1 and type 2 endothelial TJs. (**E**) Quantification of the junction type frequency in control and mutant brains. (**F**) Type 2 junction distance (nm) is increased in *Prox1^iEC-OE^* mutant brains compared with controls. (**G**) Vesicular density quantification in brain ECs, normalized to area (μm^2^). *Prox1^iEC-OE^* mutant brains displayed increased vesicular density, including luminal, cytoplasmic, and abluminal vesicles. (**H**) Representative TEM images showing vesicles in mutant samples (arrowheads). *n* = 5–6 biological samples from 3 independent experiments. Mean ± SEM, unpaired *t* test. *P < 0.05, **P < 0.001 ***P < 0.0005, ****P < 0.0001. Scale bars: 800 nm (**C** and **D**, right), 200 nm (**D**, left),100 nm (**H**).

Since impaired barrier function correlates with impaired TJ proteins, we observed a reduction in the expression of CLDN5 in the brain vasculature of *Prox1^iEC-OE^* mutants (Supplemental Figure 7B and quantification in Supplemental Figure 7D). Supporting this observation, we also found a decrease in the mRNA expression of BBB markers, such as *Cldn5*, *Tjp1*, *Cd93*, *Fgfbp1*, and *Mfsd2a*, and an increase in the expression of *Plvap* and *Cav1*, in *Prox1^iEC-OE^* compared with control brain ECs (Figure 10A). These findings demonstrate that endothelial *Prox1* expression disrupts barrier integrity in the postnatal CNS vasculature.

Given that EC β-catenin signaling is known to maintain the BBB state (48, 59–62), we observed a decrease in the expression of *Ctnnb1* as well as several effector and target genes associated with Wnt/β-catenin signaling in the mutants compared with controls (Figure 10B and Supplemental Figure 7F). Taken together with the findings from the analysis of the developing CNS vasculature, these data show that endothelial *Prox1* expression significantly downregulates Wnt/β-catenin signaling in both developing and postnatal CNS vasculature.

Recent studies show that Wnt/β-catenin signaling activates *Mfsd2a*, which limits caveolae-mediated transcytosis in CNS ECs (52, 53, 59, 63). This prompted us to examine how *Prox1* expression affects both transcellular and paracellular permeability in the postnatal CNS vasculature. To provide ultrastructural validation of these molecular findings, we performed a comprehensive transmission electron microscopy (TEM) analysis on control and mutant brains (Figure 10C). We classified TJs into 2 types: type 1 (normal) with dense protein accumulation and narrow intercellular spacing (<3 nm), and type 2 (abnormal) with wider gaps (≥3 nm) and lower protein density (Figure 10D and Supplemental Figure 7I, arrows). *Prox1^iEC-OE^* mutants displayed a significantly higher proportion of type 2 junctions than controls (Figure 10E), with significantly increased intercellular distances at these junctions (Figure 10F), indicating impaired paracellular barrier integrity. We also assessed vesicle density to evaluate transcytosis. TEM analysis revealed more vesicles in the luminal, cytoplasmic, and abluminal compartments of brain ECs in *Prox1^iEC-OE^* mutants (Figure 10G). Representative examples of vesicle types are shown in Figure 10H (arrowheads) and Supplemental Figure 7I (yellow arrowheads). These data indicate that *Prox1* overexpression enhances both paracellular and transcellular permeability in the postnatal CNS vasculature, leading to BBB breakdown.

To further investigate whether the BBB defects arise specifically from *Prox1* overexpression in the brain endothelium rather than from peripheral LECs or non-brain ECs, we used *Slco1c1-Cre^ERT2^* driver (33) to analyze *Slco1c1-Cre^ERT2^ R26-LSL-Prox1* (hereafter referred to as *Prox1^BrainEC-OE^*) mice, in which *Prox1* overexpression is restricted to CNS ECs. *Prox1^BrainEC-OE^* mutants displayed a strong induction of *Prox1* expression specifically in brain ECs, and exhibited phenotypes similar to those observed in *Prox1^iEC-OE^* mutants, characterized by enlarged cortical vessels, surface hemorrhages, and severe tracer leakage in both superficial and deep parenchymal vessels (Supplemental Figure 8). These findings confirm that *Prox1* expression in brain ECs alone is sufficient to disrupt the mature BBB and induce vascular pathology, independent of any contributions from peripheral EC defects.

### Endothelial Prox1 expression disrupts TJ integrity by directly repressing claudin-5 and Ctnnb1 expression in brain ECs.

**Figure 11:**
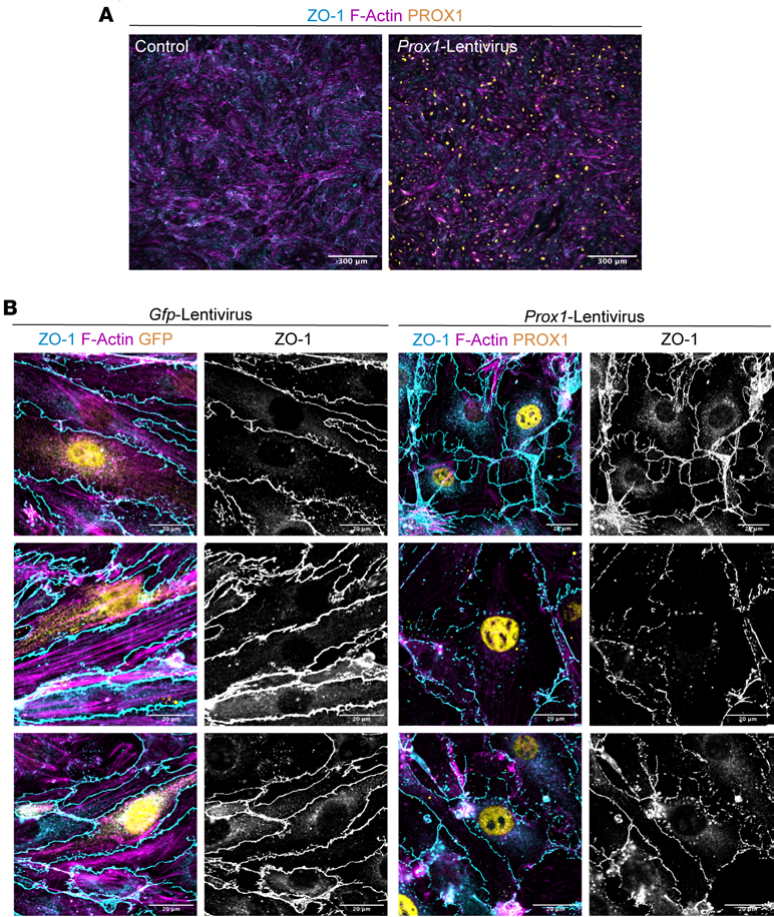
*Prox1* overexpression disrupts TJs in cultured brain ECs. (**A**) Representative images showing overall confluence of cultured bEnd.3 mouse brain ECs in control and *Prox1*-lentivirus–transduced cells, stained for ZO-1 (cyan), F-actin (magenta), and PROX1 (yellow). (**B**) High-magnification representative images of bEnd.3 cells transduced with *Gfp*- or *Prox1-*lentivirus showing ZO-1 (cyan/gray) and F-actin (magenta) with GFP or PROX1 (yellow). ZO-1 single-channel images show discontinuous cell-cell junctions and an enlarged cell shape in *Prox1*-expressing bEnd.3. Scale bars: 300 μm (**A**), 20 μm (**B**).

We next explored how PROX1 disrupts EC barrier functions. To address this question, we turned to in vitro culture experiments using a mouse brain EC line, bEnd.3 cells, known for their brain EC–specific characteristics, including the maintenance of neural stem cells (64). Importantly, previous studies demonstrated that Wnt/β-catenin signaling upregulates the expression of *Mfsd2a* while downregulating the expression of *Cav1* and *Plvap* in cultured bEnd.3 cells (59). Given that endogenous PROX1 was not detectable in bEnd.3 cells (Figure 11A and Supplemental Figure 9C), we introduced the *Prox1* or *Gfp* transgene into the cells using a lentiviral system and subsequently cultured these infected cells until they formed confluent monolayers (Figure 11A and Supplemental Figure 9, A and B). Consistent with in vivo findings, *Prox1*-overexpressing bEnd.3 cells exhibited disrupted junctional organization, as demonstrated by ZO-1 immunostaining, while control cells formed characteristic continuous junctions (Figure 11B; 3 representative images each for bEnd.3 cells expressing *Prox1* or *Gfp*).

**Figure 12:**
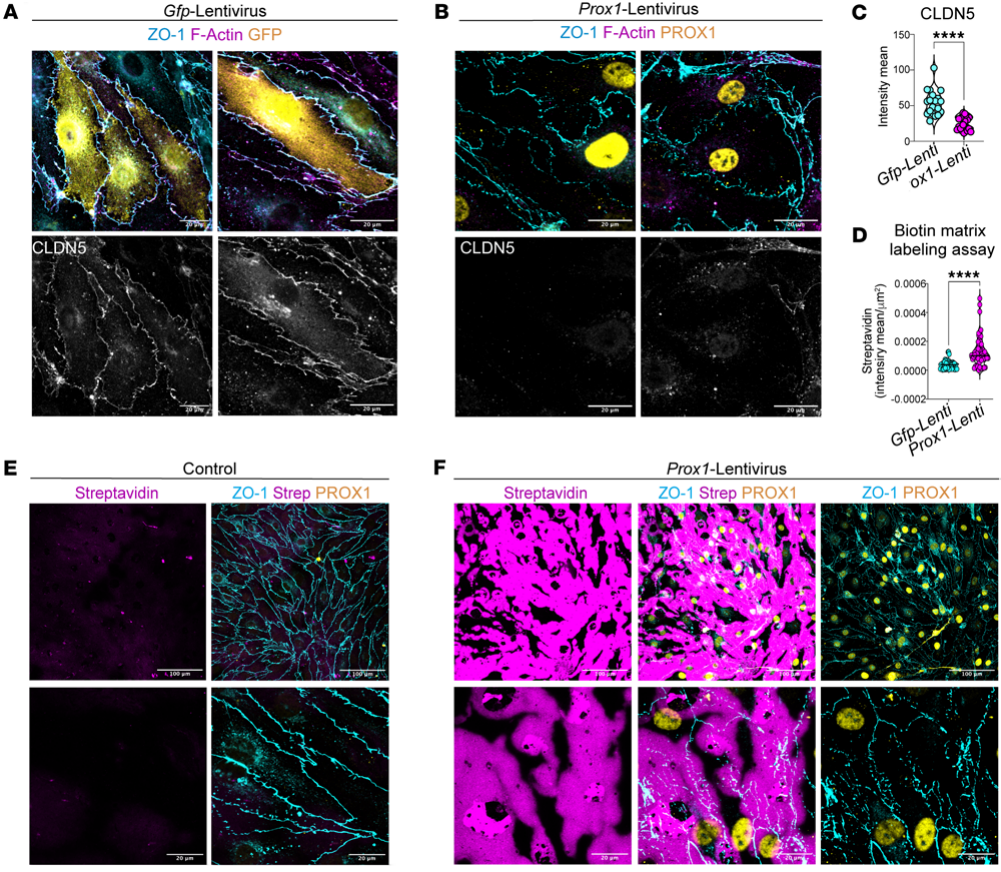
*Prox1* represses *Cldn5* and increases monolayer permeability in cultured brain ECs. (**A** and **B**) Representative images of bEnd.3 cells transduced with *Gfp-* or *Prox1-*lentivirus, stained for ZO-1 (cyan/gray), CLDN5 (magenta/gray), and GFP or PROX1 (yellow). Bottom panels show CLDN5 in grayscale. (**C**) Quantification of CLDN5 intensity. *n* = 20 fields of view from 4 independent experiments. Mean ± SEM, unpaired *t* test. (**D**–**F**) Biotin matrix assay. (**D**) Quantification of streptavidin-positive area/μm^2^. *n* > 50 fields of view from 5 independent experiments. Mean ± SEM, unpaired *t* test. ****P < 0.0001. (**E** and **F**) Representative images of control (**E**) and *Prox1*-expressing cells (**F**) showing streptavidin (magenta), ZO-1 (cyan), and PROX1 (yellow). Scale bars: 20 μm (**A** and **B**; and **E** and **F**, bottom panels), 100 μm (**E** and **F**, top panels).

To assess the impact of PROX1 on BBB-selective TJ components, we examined the expression of CLDN5, a critical TJ protein required for BBB integrity. Immunostaining revealed a marked reduction of CLDN5 in *Prox1*-overexpressing cells, at both the membrane and cytoplasmic levels, compared with controls (Figure 12, A and B), and this reduction was quantitatively significant (Figure 12C). Importantly, a biotinylation-based monolayer permeability assay demonstrated compromised barrier function in *Prox1*-overexpressing cultures, supporting a functional consequence of CLDN5 loss (Figure 12, E and F, Supplemental Figure 9D, and quantification in Figure 12D). Notably, we also observed abnormal cell-cell junctions in most primary rat brain microvascular ECs (RBMVECs) expressing *Prox1* (Supplemental Figure 9, E and F). Collectively, these in vitro studies present compelling evidence of abnormal TJs due to the endothelial *Prox1* expression in brain ECs.

**Figure 13:**
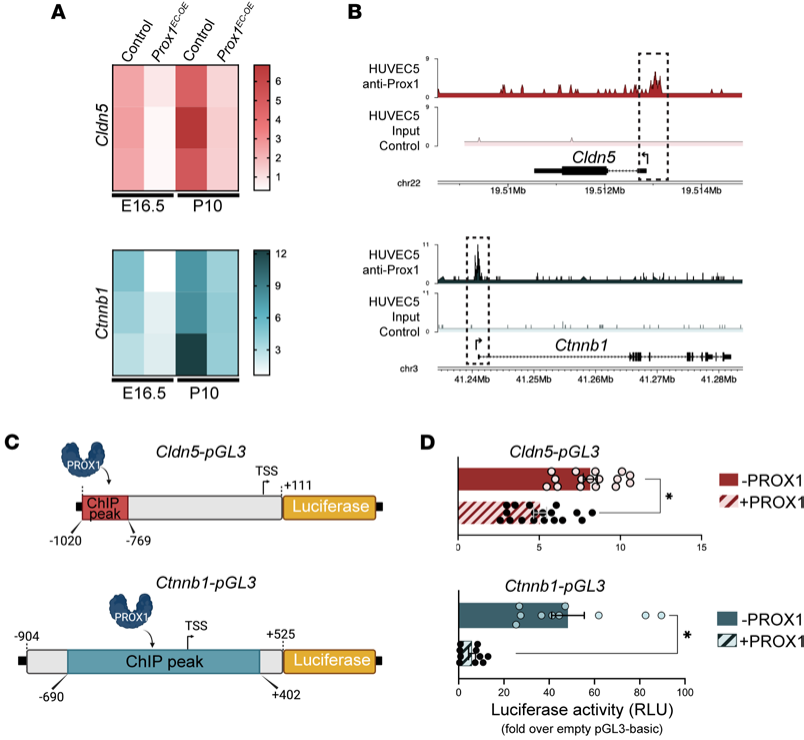
*Prox1* directly represses *Cldn5* and *Ctnnb1* transcription. (**A**) Heatmaps showing relative mRNA expression levels of *Cldn5* (top) and *Ctnnb1* (bottom) in brain ECs from control and *Prox1^iEC-OE^* mice at E16.5 and P10, as measured by quantitative reverse transcription PCR. (**B**) A published whole-genome ChIP-Seq study using anti-PROX1 antibody in HUVECs expressing *Prox1* (69) reveals the presence of PROX1-binding sites at the promoter of *Cldn5* and *Ctnnb1*. These data are available online through the Gene Expression Omnibus (GEO) under reference GSE71230. (**C** and **D**) Luciferase reporter assay. (**C**) Schematics of luciferase reporter constructs containing PROX1-bound enhancer regions at *Cldn5* (top) and *Ctnnb1* (bottom) promoters, cloned upstream of the luciferase gene in the pGL3-basic vector. Created in BioRender (Gonzalez S, 2025, https://BioRender.com/kdo2upo). (**D**) Luciferase assays in bEnd.3 cells transfected with the *Cldn5*- or *Ctnnb1*-pGL3 and empty (–PROX1) or *Prox1*-expressing (+PROX1) vectors. PROX1 reduced reporter activity for both genes. *n* = 10–18 wells from 3–4 independent experiments. Mean ± SEM, unpaired *t* test. *P < 0.05.

The foregoing in vivo and in vitro studies demonstrate that *Prox1* expression in brain ECs leads to a decrease in the mRNA expression of *Cldn5* and a reduction of both junctional and cytoplasmic CLDN5 in brain ECs (Figure 12, A–C). Considering prior reports suggesting that PROX1 functions as a transcriptional repressor in neural progenitors (65), hepatocytes (66), and cancers (67, 68), it was plausible that PROX1 regulated CLDN5 expression through direct transcriptional suppression of the *Cldn5* gene. Analysis of a published whole-genome chromatin immunoprecipitation sequencing (ChIP-Seq) using an anti-PROX1 antibody in human umbilical vein ECs (HUVECs) expressing *Prox1* revealed the presence of PROX1-binding sites at the promoter of *Cldn5* and *Ctnnb1* genes (69) (Figure 13B). Given that endothelial *Prox1* expression decreased the mRNA levels of *Cldn5* and *Ctnnb1* (Figure 13A), PROX1 may regulate the transcription of these genes.

**Figure 14:**
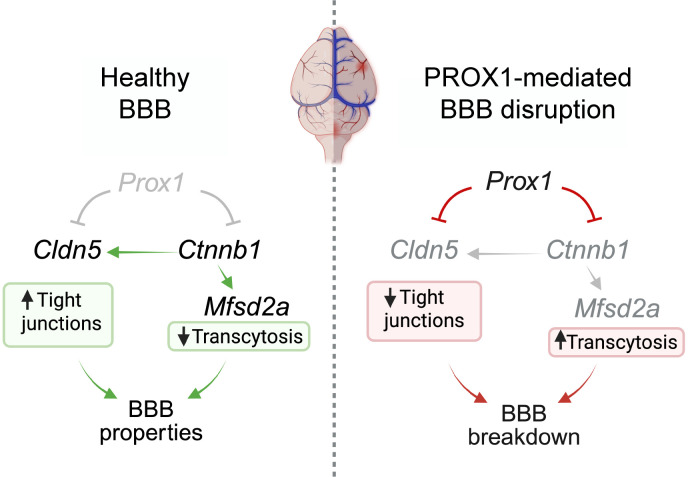
PROX1-mediated BBB disruption. Schematic model showing that in homeostatic brain ECs (left), absence of *Prox1* allows *Cldn5* and *Ctnnb1* expression, promoting TJs and *Mfsd2a*-dependent suppression of transcytosis, thereby maintaining BBB integrity. In contrast, aberrant *Prox1* expression in brain ECs (right) represses *Cldn5* and *Ctnnb1*, reducing TJs and increasing transcytosis, leading to BBB breakdown. Created in BioRender (Gonzalez S, 2025, https://BioRender.com/kdo2upo).

To functionally validate this, we cloned putative PROX1-bound promoter/enhancer regions from *Cldn5* and *Ctnnb1* upstream of a luciferase reporter gene (Figure 13C). Cotransfection of these luciferase constructs with either control or *Prox1*-expressing vectors into bEnd.3 cells demonstrated that PROX1 significantly repressed transcriptional activity from both *Cldn5* and *Ctnnb1* regulatory regions (Figure 13D). These findings provide direct mechanistic evidence that *Prox1* suppresses the expression of key BBB regulators by acting as a transcriptional repressor. Taking these findings together, we propose a model in which aberrant *Prox1* expression in brain ECs compromises BBB integrity by repressing *Cldn5* and *Ctnnb1*, leading to disrupted TJs and enhanced transcytosis, in part due to secondary downregulation of *Mfsd2a* (Figure 14). Supporting this, PROX1 ChIP-Seq data identified a binding peak at the *Cd93* promoter but not at the *Mfsd2a* locus (Supplemental Figure 10A), suggesting that the reduced *Mfsd2a* expression observed in *Prox1*-overexpressing ECs is likely an indirect consequence of *Ctnnb1* suppression. These results demonstrate that PROX1 compromises BBB integrity by directly repressing key genes required for TJ formation and barrier maintenance, including *Cldn5* and *Ctnnb1*, and establish a mechanistic link between PROX1 abnormal expression and endothelial barrier dysfunction in the CNS.

## Discussion

The CNS parenchyma is immune-privileged because of unique barriers like the BBB and lack of lymphatic vasculature. In conditions like brain tumors and AVMs, which compromise vascular integrity, LEC markers like PROX1 and the vascular permeability marker PLVAP increase. Our findings demonstrate that PROX1 compromises BBB integrity by downregulating TJ proteins and Wnt/β-catenin signaling, leading to enhanced paracellular and transcellular leakage. This occurs without forming conventional lymphatic vasculature, instead creating a hybrid blood-lymphatic state during embryonic but not postnatal stages, highlighting the inhibitory role of PROX1 in BBB development and maintenance. Mechanistically, PROX1 functions as a negative regulator of BBB-associated genes and Wnt/β-catenin signaling in CNS ECs, explaining its destabilizing effects on barrier function.

Given that the human scRNA-Seq analysis suggests a potential link between the LEC marker expression and the BBB disruption, there is a technical limitation: in GBM, *PROX1* expression appeared scattered rather than confined to a defined EC cluster, which may reflect tumor heterogeneity or integration across studies. Similarly, the complete segregation of disease and control ECs could partly represent technical differences rather than biological ones. Thus, although LEC-associated transcripts such as PROX1, LYVE1, and FLT4 were consistently upregulated in CNS pathologies, increased expression alone does not necessarily indicate LEC differentiation. This highlights the need for cautious interpretation and additional in vivo validation.

Embryonic *Prox1* induction triggers the transformation of blood vessels into hybrid blood-lymphatic vessels, similar to Schlemm’s canal ECs, rather than conventional lymphatic vessels, within the brain parenchyma. In contrast, postnatal induction of *Prox1* does not result in a hybrid phenotype, as *Prox1^iEC-OE^* brain ECs fail to upregulate FLT4/VEGFR3, underscoring the stage-specific restrictions imposed by the CNS microenvironment. Given that VEGF-C/VEGFR3 signaling is crucial for Schlemm’s canal development (43, 44), lower VEGFR3 expression in the postnatal CNS vasculature may be insufficient to induce a hybrid blood-lymphatic phenotype. Because *Flt4*/*VEGFR3* is a direct target gene of PROX1 (70), the postnatal CNS parenchyma likely enforces a microenvironment that suppresses FLT4/VEGFR3 upregulation. Detailed molecular mechanisms underlying the suppression of LEC markers, such as FLT4/VEGFR3, remain to be elucidated.

Endothelial *Prox1* expression leads to vascular leakage and BBB disruption when induced during both embryonic and postnatal stages, reinforcing its inhibitory role in barrier integrity. This is associated with reduced expression of TJ proteins such as CLDN5 and ZO-1, along with the induction of PLVAP, a marker of high-permeability vasculature. Although TEM analysis did not reveal discontinuous junctions or fenestrations in *Prox1^iEC-OE^* mutant capillaries, cultured bEnd.3 cells expressing *Prox1* displayed disrupted junctions. The apparent stability of capillary junctions in *Prox1^iEC-OE^* mutants in vivo may reflect protective pericyte coverage, which is absent in cultured brain ECs. In addition, our findings indicate that *Prox1* expression leads to the upregulation of transcytosis, as indicated by reduced expression of *Mfsd2a*, a lipid transporter that limits transcytosis in the BBB, and elevated expression of *caveolin*/*CAV1*, accompanied by an increased number of endothelial vesicles. Given that *Mfsd2a* expression is transcriptionally regulated by Wnt/β-catenin signaling in both in vivo (52, 53, 59, 63) and cultured bEnd.3 cells (53), *Prox1* indirectly upregulates transcytosis by downregulating Wnt/β-catenin signaling. Supporting this conclusion, our extensive TEM analysis revealed a significant increase in structurally abnormal type 2 junctions — defined by widened inter-endothelial gaps and reduced electron density — as well as elevated vesicle density in *Prox1^iEC-OE^* mutants. These data confirm that PROX1 promotes both paracellular and transcellular leakage mechanisms at the ultrastructural level.

As impaired EC β-catenin signaling increases paracellular and intercellular BBB permeability (48, 59–62), endothelial *Prox1* expression leads to BBB disruption by inhibiting the *Ctnnb1* or *Cldn5* gene in ECs. How does PROX1 function as a transcriptional repressor in brain ECs? In hepatocytes, PROX1 interacts with the class I histone deacetylase HDAC3 to cooperatively repress gene transcription critical for maintaining lipid homeostasis (66). In colorectal cancer cells, PROX1 interacts with HDAC1 in the nucleosome remodeling and deacetylase (NuRD) complex to suppress the Notch pathway (67). Indeed, HDAC2 mediates transcriptional regulation of BBB genes during BBB formation and maintenance (71). Thus, it is plausible that PROX1 may interact with class I histone deacetylases such as HDAC2 to suppress the expression of *Ctnnb1* or *Cldn5* in brain ECs.

Our study demonstrates that while the CNS establishes a non-permissive microenvironment for the development and growth of conventional lymphatic vasculature under physiological conditions, endothelial *Prox1* expression is sufficient to trigger vascular malformations and BBB disruption. These findings indicate that strict suppression of *Prox1* expression in CNS ECs is necessary to preserve BBB integrity. Similar principles operate in non-CNS organs, where *Prox1* suppression is essential for maintaining blood-lymphatic segregation. For instance, deficiency in *folliculin* (*FLCN*), the tumor suppressor gene responsible for Birt-Hogg-Dubé syndrome, leads to endothelial *Prox1* expression in veins and aberrant blood-lymphatic connections (39). In zebrafish, vascularization of the anal fin involves transdifferentiation of lymphatic vessels into blood vessels, with *Sox17* acting to suppress *Prox1* expression to enable the LEC-to-BEC transition (72).

Further studies are necessary to clarify the fundamental mechanisms underlying *Prox1* suppression in brain ECs and the lack of lymphatic vessels within the CNS parenchyma. Dysregulation of this suppression is likely a contributing factor to BBB dysfunction in various CNS diseases. Understanding the molecular links between *Prox1* regulation and barrier disruption in disease states could lead to new therapeutic strategies. These could include temporarily opening the BBB to enhance the delivery of therapeutic agents to the brain or restoring the barrier integrity in disease conditions.

## Methods

### Sex as a biological variable.

In this study, sex was not included as a biological variable in embryos and neonates owing to the technical challenges associated with distinguishing sex at late embryonic and early postnatal stages.

### Mice.

The following mice (*Mus musculus*) were used in this study: We obtained C57BL/6J mice and CD-1 mice from The Jackson Laboratory and Charles River Laboratories, respectively. We obtained *Cadh5-BAC-Cre^ERT2^* mice (40) from the Yoshiaki Kubota laboratory at Keio University (Shinjuku, Tokyo, Japan), *Prox1-GFP BAC* mice (37) from the Young-Kwon Hong laboratory at the University of Southern California (Los Angeles, California, USA), and *Slco1c1-Cre^ERT2^* mice (33) from the Injune Kim laboratory at Korea Advanced Institute of Science & Technology (Daejeon, South Korea). *Rosa26-LSL-Prox1* mice were generated in the Mukouyama laboratory and the National Heart, Lung, and Blood Institute (NHLBI) Transgenic Core. For timed pregnancies, the morning of the vaginal plug was considered E0.5. Tamoxifen (Sigma-Aldrich) was administered intraperitoneally at E13.5 (1.5–3 mg) for embryonic induction or at P7–P10 (0.5 mg) for postnatal induction. Embryos were harvested at E16.5 and pups at P10–P13.

### Generation of R26-LSL-Prox1 mice.

The generation of *Rosa26-LSL-Prox1* mice was previously described (39). Briefly, a mouse *Prox1* coding sequence with 5′ FLAG tag was knocked into the mouse *Rosa26* locus using the CRISPR/Cas9 method in the NHLBI Transgenic Core. The *R26-loxP-STOP-loxP-Prox1* construct was co-microinjected along with *Cas9* mRNA and sgRNA into the pronuclei of fertilized mouse eggs. After culturing of the injected embryos overnight, embryos that had reached the 2-cell stage of development were implanted into the oviducts of pseudopregnant foster mothers.

### scRNA-Seq analysis of publicly available datasets.

To evaluate lymphatic marker gene expressions, publicly available scRNA-Seq datasets were utilized. For the GBM datasets, raw count matrices from publicly available Gene Expression Omnibus (GEO) datasets GSE162631, GSE173278, and GSE184357 (28–30) were processed using the standard Seurat workflow. Among these datasets, GSE162631 was deposited as unfiltered (raw) count matrices, whereas GSE173278 and GSE184357 were available in pre-filtered form; the latter were used as provided for downstream analyses. For GSE162631, raw count matrices from 8 samples (R1_N, R1_T, R2_N, R2_T, R3_N, R3_T, R4_N, and R4_T) were imported. Low-quality cells — defined as those with fewer than 200 detected genes or more than 10% mitochondrial gene content — were excluded. Each sample was integrated using reciprocal PCA–based anchor identification and Seurat’s IntegrateData function. The integrated dataset underwent PCA and uniform manifold approximation and projection (UMAP) (dims = 1:30), and clustering was conducted at a resolution of 0.5. Cell annotation was performed based on marker genes reported in the original publication (30), and UMAP plots were generated accordingly. GSE173278 and GSE184357 were processed using the filtered count matrices and associated metadata files. For both datasets, a Seurat object was constructed, and cell-level metadata were incorporated. Dimensionality reduction was performed via PCA followed by UMAP (dims = 1:20). UMAP plots were generated using the provided cell annotations. ECs were subset from each of the 3 datasets (GSE162631, GSE173278, and GSE184357) using canonical marker genes *CLDN5*, *VWF*, and *CD34*. Each endothelial dataset was independently normalized and subjected to variable feature selection using the variance-stabilizing transformation (vst) method. Shared highly variable features (*n* = 2,000) were identified across datasets using the SelectIntegrationFeatures function. Subsequently, each dataset was scaled and underwent PCA using the identified features. Integration anchors were computed via FindIntegrationAnchors, and the 3 datasets were integrated using Seurat’s IntegrateData function. The integrated Seurat object was scaled, and dimensionality reduction was performed using PCA and UMAP (dims = 1:30). Clustering was conducted at a resolution of 0.7. Annotation of tumor core and periphery was performed using the 5 clusters defined in the original paper (30), followed by UMAP visualization. Additionally, a heatmap of marker genes characterizing these clusters was generated.

For brain metastasis, the dataset was downloaded from Brain TIME (Johanna Joyce Laboratory, https://joycelab.shinyapps.io/braintime/). For AVMs (32), the dataset was downloaded from the UCSC Cell Browser (https://adult-brain-vasc.cells.ucsc.edu). Both datasets contained pre-selected EC populations, which were directly used for downstream analysis. Accompanying metadata were used to annotate EC subtypes, and UMAP plots were generated accordingly.

For all 3 disease datasets (GBM, brain metastasis, and AVMs), UMAP plots were visualized using either Seurat’s default plotting functions, scCustomize package in R (73), or the Scanpy package in Python (https://scanpy.readthedocs.io/en/stable/). To calculate average gene expressions of lymphatic markers (*PROX1*, *LYVE1*, and *FLT4*) and *PLVAP*, the AverageExpression function in Seurat was used.

### Histology and immunofluorescence.

Embryos and neonates were fixed in 4% paraformaldehyde (PFA) overnight at 4°C, cryoprotected in sucrose, embedded in Tissue-Tek OCT Compound (Sakura), and sectioned. Cryosections were permeabilized (0.5% Triton X-100/PBS), blocked (10% goat serum/0.1% Triton X-100/PBS or 1% bovine serum albumin/0.1% Triton X-100/PBS), and incubated with primary antibodies (1:100 to 1:200) overnight at 4°C, followed by fluorophore-conjugated secondary antibody incubation (1:300 to 1:500). Negative controls omitted primary antibodies. The antibodies used are listed in Supplemental Table 1.

### Tissue clearing and whole-mount staining.

The CUBIC protocol was used for tissue clearing as previously described (74, 75). Tissues were incubated in CUBIC reagent-1 [25 wt% urea, 25 wt% *N*,*N*,*N*′,*N*′-tetrakis(2-hydroxypropyl) ethylenediamine, and 15% (vol/vol) Triton X-100] for 1–2 days at room temperature with rotation. Tissues were briefly washed in PBS, blocked, and stained with primary (1:300) and secondary (1:500) antibodies. After whole-mount immunostaining, tissues were balanced with sucrose (20%) and incubated in CUBIC reagent-2 [50 wt% sucrose, 25 wt% urea, 10 wt% 2,2′,2′-nitrilotriethanol, and 0.1% (vol/vol) Triton X-100] at room temperature. Cleared tissues were mounted in CUBIC reagent-2 and imaged on a Leica TCS SP5 microscope.

### Flow cytometry and FACS.

Brains were isolated in cold HBSS medium (Thermo Fisher Scientific), minced and digested (0.05% DNase I, 0.1% collagenase, 0.3% dispase, in Leibovitz’s L-15 medium [Thermo Fisher Scientific]), and filtered (70 μm). Negative selection for TER119^+^ and CD45^+^ cells was performed with magnet beads. Endothelial cells (DAPI^–^TER119^–^CD45^–^CD140b^–^CD31^+^LYVE1^+/–^) and pericytes (DAPI^–^TER119^–^CD45^–^CD140b^+^CD31^–^) were analyzed or sorted using BD instruments (BD FACSDiscover S8 Sorter, BD FACSAria Fusion Flow Cytometer, and BD FACSymphony S6 Cell Sorter). Unstained samples, single-color staining, and fluorescence minus one (FMO) were used to establish the proper compensation and gating. Antibodies used for cytometry are listed in Supplemental Table 1. Data were analyzed using FlowJo software (BD Biosciences).

### Quantitative reverse transcription PCR.

RNA was extracted from FACS-isolated ECs using PicoPure RNA Isolation Kit (Thermo Fisher Scientific), reverse-transcribed using SuperScript III Reverse Transcriptase (Thermo Fisher Scientific), and amplified by quantitative reverse transcription PCR with Power SYBR Green Master Mix 2X (Roche). The general cycling conditions were as follows: 1 initial hold for 3 minutes at 95°C, followed by 40 cycles of 10-second denaturation (95°C) and 45-second annealing/extension at 60°C. Gene expression was normalized to *Gapdh*.

Primer sequences are listed in Supplemental Table 2.

### BBB permeability assays.

For embryonic assays, 3 kDa dextran Texas red (Invitrogen) was injected into the left ventricle (10 μg in PBS), and embryos were incubated in HBSS for 5 minutes and fixed with 10% PFA/PBS for 2 hours at room temperature. Dissected brains were processed for cryosections or tissue clearing with subsequent whole-mount immunostaining.

For postnatal assays, pups were injected intraperitoneally with 3 kDa dextran Texas red (Invitrogen) per 20 g mouse or 100 μg 1 kDa cadaverine (Thermo Fisher Scientific) per 20 g mouse, as previously reported (76). After 2 hours, pups were euthanized, and brain tissues were harvested for fixation and posterior analysis. Leakage was determined by making a mask of the vasculature area using the PECAM1 or EMCN channel, then assessing the dextran or cadaverine signal outside of the vasculature.

### Cell culture and lentiviral transduction.

bEnd.3 cells (ATCC) and rat brain microvascular ECs (RBMVECs; Cell Applications) were cultured in recommended media. For lentiviral transduction, cells were seeded into 12-well glass chamber slides (ibidi) coated with 10 μg/mL fibronectin (MilliporeSigma) or Attachment Factor Solution (Cell Applications). Once cells reached about 60%–70% confluence, cell medium was removed and fresh cell medium containing 1 mg/mL Polybrene (VectorBuilder) and *Gfp-* or *Prox1-*expressing lentivirus was added (MOI 5–10). Culture medium was changed after 48 hours. Cells were fixed with 4% PFA when they reached a confluent monolayer. Immunostaining was performed as described above. Cells were permeabilized (0.1% Triton X-100/PBS), blocked (1% bovine serum albumin/0.1% Triton X-100/PBS), and stained with antibodies (Supplemental Table 1). Confocal imaging was carried out on a Leica TCS SP5 microscope using a ×63 oil objective.

### Biotin matrix labeling assay.

Labeling of biotinylated matrix was assessed as previously described (77). Briefly, fibronectin (0.1 mg/mL) was biotinylated (0.5 mM EZ-Link Sulfo-NHS-LC-Biotin, Thermo Fisher Scientific) and coated (5 μg/mL) onto glass chamber slides (ibidi). Confluent bEnd.3 cells were transduced with lentivirus as described above. After 48 hours, cells were incubated with 25 μg/mL Alexa Fluor 488–conjugated streptavidin (Invitrogen) to detect matrix-bound biotin. Cells were immediately fixed and processed for imaging.

### Cloning of the murine Cldn5 and Ctnnb1 enhancer/promoter region.

The *pGL3-basic* luciferase reporter vector (Promega) was purchased from Addgene and used as the backbone for enhancer activity assays. Two mouse genomic enhancer regions were selected: a *Cldn5* enhancer region spanning –1020/+111 bp relative to the transcription start site (TSS) as previously described (78), and a *Ctnbb1* enhancer region spanning –904/+525 bp relative to the TSS. These DNA fragments were synthesized by GenScript and subcloned into the multiple cloning site of the *pGL3-basic* vector using MluI and HindIII restriction enzymes, upstream of the firefly luciferase gene (*luc2*). The resulting constructs, *Cldn5-pGL3* and *Ctnnb1-pGL3*, were sequence-verified and used for subsequent luciferase reporter assays.

### Luciferase reporter assay.

bEnd.3 mouse brain ECs were seeded in 24-well plates at a density of 4 × 10^5^ cells per well. When they reached confluence, transient transfection was performed with Lipofectamine 3000 (Thermo Fisher Scientific) following the manufacturer’s protocol. Each transfection contained 300 ng of enhancer construct DNA (*Cldn5-pGL3* or *Ctnnb1-pGL3*), 300 ng of expression plasmid (*pcDNA3.1* empty vector or *pcDNA3.1-mProx1*), and 80 ng of the internal control plasmid *pRL-TK* (Promega). After 24 hours, the culture medium was replaced with fresh medium. Luciferase activity was measured using the Dual-Glo Luciferase Assay System (Promega), according to the manufacturer’s instructions. Both firefly and Renilla luciferase activities were quantified using a luminometer, with each well measured in triplicate. Firefly luciferase activity (reporter) was normalized to Renilla luciferase reporter (internal control), and results were expressed as relative light units (RLU). Normalized enhancer signals (with or without *Prox1* coexpression) were further normalized to the signal obtained with the empty *pGL3-basic* vector, which lacks enhancer insert. Final values represent fold change over *pGL3-basic* background activity.

### Transmission electron microscopy.

Postnatal brains were harvested and fixed in a 0.1 M sodium cacodylate–buffered mixture (2.5% glutaraldehyde/4% PFA) for 2 hours at room temperature followed by overnight incubation in 4% PFA at 4°C. The next day, tissues were washed in 0.1 M sodium cacodylate buffer and then cut in 200-μm-thick free-floating sections using a vibratome. Sections were then postfixed in 2% osmium tetroxide and 1.5% potassium ferrocyanide and stained overnight in 1% UA. The following day, samples were dehydrated in graded ethanol series, infiltrated with resin (EMbed-812, Electron Microscopy Sciences), and baked at 60°C for 48 hours. Ultrathin sections (65–70 nm) were cut on an ultramicrotome (Leica EM UC7), and digital micrographs were acquired with a JOEL JEM 1200 EXII (80 kV) equipped with an AMT XR-60 digital camera.

### Statistics.

Data are from 3 or more independent experiments unless stated. Exact biological replicate numbers are given in figure legends. Statistical analyses were performed with GraphPad Prism 9. Normality was assessed by Shapiro-Wilk test; unpaired 2-tailed *t* tests were applied for normality distributed data. Data are shown as mean ± SEM. *P* < 0.05 was considered significant. For all the images included throughout the article, the most representative examples reflecting the typical phenotype were selected.

### Study approval.

All animal procedures were approved by the National Heart, Lung, and Blood Institute (NHLBI) Animal Care and Use Committee in accordance with NIH research guidelines for the care and use of laboratory animals.

### Data availability.

All data in the article are included in the Supporting Data Values file.

## Author contributions

SGH conducted all the experiments, and also contributed to the conceptualization, writing, and editing of the manuscript. YS, WL, Chang Liu, and Chengyu Liu were responsible for generating and conducting the primary characterization of *R26-LSL-Prox1* mice. RS performed the analysis of publicly available scRNA-Seq data. ZAS performed the TEM imaging. SJ and YK provided valuable reagents and technical advice. YM contributed through project supervision, discussion, and writing and editing of the manuscript.

## Funding support

This work is the result of NIH funding, in whole or in part, and is subject to the NIH Public Access Policy. Through acceptance of this federal funding, the NIH has been given a right to make the work publicly available in PubMed Central.

- Intramural Research Program of the NHLBI, NIH (HL006115-14 to YM).
- NHLBI Lenfant Biomedical Fellowship to SGH.

## Supplementary Material

Supplemental data

Supplemental video 1

Supplemental video 2

Supporting data values

## Version 1 01/09/2026

Electronic publication
