# Supplementary material for: Endothelial PROX1 induces blood-brain barrier disruption in the central nervous system
Source: JCI Insight. 2026 Jan 9;11(1):e187716. doi: 10.1172/jci.insight.187716 (PMC12890493; doi:10.1172/jci.insight.187716)
Supplement: Supplemental data [file jciinsight-11-187716-s228.pdf]

SUPPLEMENTAL INFORMATION

ENDOTHELIAL PROX1 INDUCES BLOOD-BRAIN BARRIER DISRUPTION IN THE CENTRAL NERVOUS SYSTEM

Sara González-Hernández, Ryo Sato, Yuya Sato, Chang Liu, Wenling Li, Chengyu Liu, Zulfeqhar A Syed, Sadhana Jackson, Yoshiaki Kubota, Yoh-suke Mukouyama

Supplemental Figures

Supplemental Figure 1: Endothelial subclusters from glioblastoma, brain metastasis and brain arteriovenous malformations scRNA-seq datasets.
Supplemental Figure 2: Lack of *Prox1* expression in CNS vasculature.
Supplemental Figure 3: Absence of lymphatic vessels in the brain parenchyma.
Supplemental Figure 4: Characterization of *Prox1*$^{iEC\text{-}OE}$ embryos.
Supplemental Figure 5: Endothelial *Prox1* induces aberrant lymphatic vasculature in non-CNS tissues.
Supplemental Figure 6: Blood-brain barrier disruption in E16.5 *Prox1*$^{iEC\text{-}OE}$ embryos.
Supplemental Figure 7: Postnatal induction of *Prox1* disrupts the mature BBB without inducing a hybrid blood-lymphatic phenotype.
Supplemental Figure 8: Brain endothelial-specific *Prox1* overexpression disrupts BBB integrity.
Supplemental Figure 9: PROX1 disrupts endothelial junction integrity in cultured brain ECs.
Supplemental Figure 10: PROX1 represses *Cd93* but not *Mfsd2a* expression.

Supplemental Movies

Supplemental Movie 1, related to Figure 8. Imaging of vascular permeability in E16.5 control brain.
Supplemental Movie 2, related to Figure 8. Imaging of vascular permeability in E16.5 *Prox1*$^{iEC\text{-}OE}$ mutant brain.

Supplemental Tables

Supplemental Table 1: List of antibodies
Supplemental Table 2: List of primers for qRT- PCR

Supplemental Figure 1

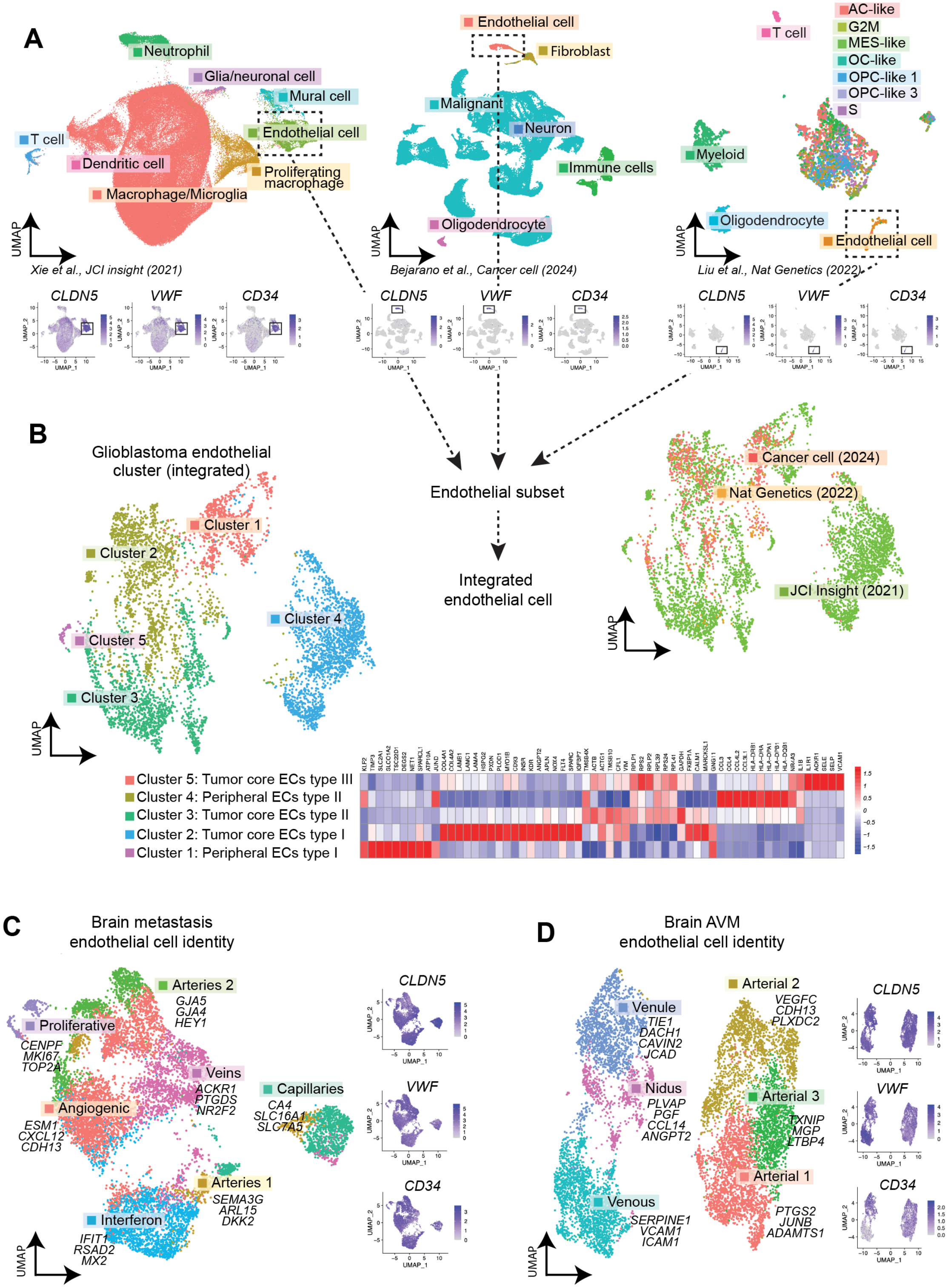

Supplemental Figure 1. Endothelial subclusters from glioblastoma, brain metastasis and brain arteriovenous malformations scRNA-seq datasets.

(**A**) UMAP of scRNA-seq data from three glioblastoma datasets (29-31), showing all cell populations. ECs were identified by canonical markers (*Cldn5*, *Vwf*, *Cd34*) or original annotation metadata, extracted from each dataset, and integrated. Bottom right: integrated EC population.
(**B**) Integrated glioblastoma ECs forming five clusters (per original study, (31)) with heatmap of cluster-specific markers.
(**C**) UMAP of EC subclusters from brain metastasis datasets (29) with representative markers; right, expression of canonical EC markers.
(**D**) UMAP of EC subclusters from AVM datasets (28) with representative markers; right, expression of canonical EC markers.

Supplemental Figure 2

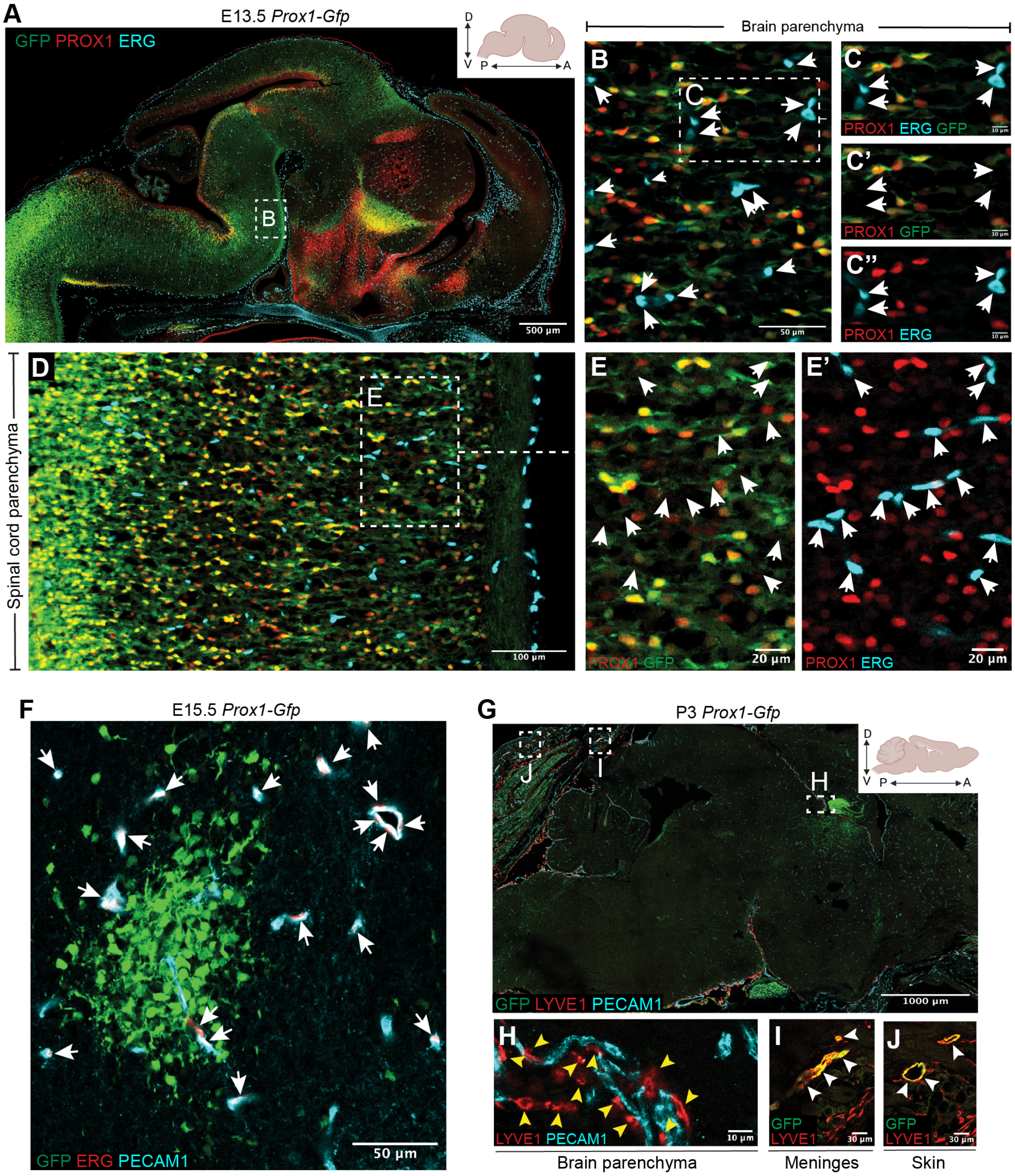

Supplemental Figure 2. Lack of *Prox1* expression in CNS vasculature.
(A-E) A sagittal view of the brain (**A-C**) and spinal cord (**D-E**) parenchyma from E13.5 *Prox1-Gfp* BAC transgenic reporter embryos labeled with PROX1 (red) and ERG (cyan). The boxed regions in (**A**, **B**, and **D**) are magnified in (**B**, **C-C''**, and **E-E'**), respectively. Arrows indicate ERG^+ EC nuclei in (**B**, **C-C''** and **E-E'**). These cells do not co-localize with PROX1 and Prox1-GFP. **(F)** Brain parenchyma of E15.5 *Prox1-Gfp* embryos labeled with PECAM1 (cyan) and ERG (red). Arrows indicate $ERG^+/PECAM1^+$ ECs. These cells do not co-localize with Prox1-GFP. **(G-J)** A sagittal view of a postnatal brain section (P3) labeled with LYVE1 (red) and PECAM1 (cyan). The boxed regions in (**G**) are magnified in (**H-J**). Yellow arrowheads in (**H**) indicate $LYVE1^+/PECAM1^-/Prox1\text{-}GFP^-$ macrophages. Arrowheads in (**I** and **J**) indicate $PECAM1^+/LYVE1^+/Prox1\text{-}GFP^+$ lymphatic vessels in the meninges and skin, respectively. Scale bars: 1000 μm (**G**), 500 μm (**A**), 100 μm (**D**), 50 μm (**B, F**), 30 μm (**I, J**), 20 μm (**E**), 10 μm (**C, H**).

Supplemental Figure 3

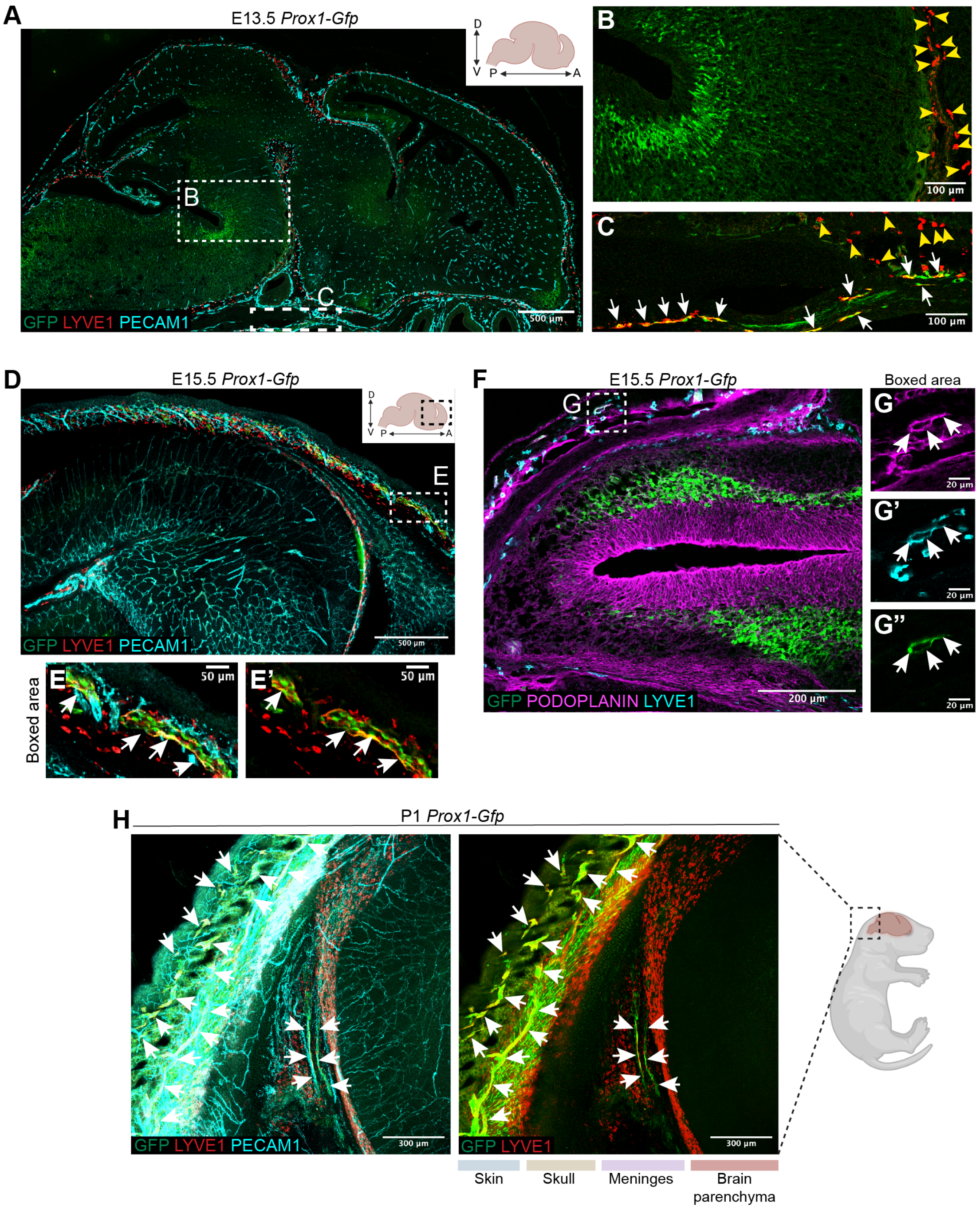

Supplemental Figure 3. Absence of lymphatic vessels in the brain parenchyma.
(A-C) Sagittal sections of E13.5 *Prox1-Gfp* heads labeled with LYVE1 (red) and PECAM1 (cyan). The boxed regions in (**A**) are magnified in (**B** and **C**). Arrows in (**C**) indicate PECAM1$^+$/LYVE1$^+$/Prox1-GFP$^+$ lymphatic vessels outside of the brain parenchyma; yellow arrowheads in (**B** and **C**) mark LYVE1$^+$/PECAM1$^-$/Prox1-GFP$^-$ macrophages.
(D-G) Sagittal sections of E15.5 *Prox1-Gfp* heads labeled with LYVE1 and PECAM1 (**D-E**) or Podoplanin and LYVE1 (**F-G**). The boxed regions in (**D** and **F**) are magnified in (**E-E'**) and (**G-G"**), respectively. Arrows indicate PECAM1$^+$/LYVE1$^+$/Prox1-GFP$^+$ lymphatic vessels (**E-E'**) and Podoplanin$^+$/LYVE1$^+$/Prox1-GFP$^+$ Lymphatic
vessels (**G-G")** in skin near the skull.
(H) A sagittal view of whole-mount immunostaining of P1 *Prox1-Gfp* head labeled with PECAM1 (cyan) and LYVE1 (red). Arrows indicate PECAM1$^+$/LYVE1$^+$/Prox1-GFP$^+$ lymphatic vessels in meninges and skin. Scale bars: 500 μm (**A, D**), 300 μm (**H**), 200 μm (**F**), 100 μm (**B**, **C**), 50 μm (**E-E'**), 20 μm (**G-G"**).

Supplemental Figure 4

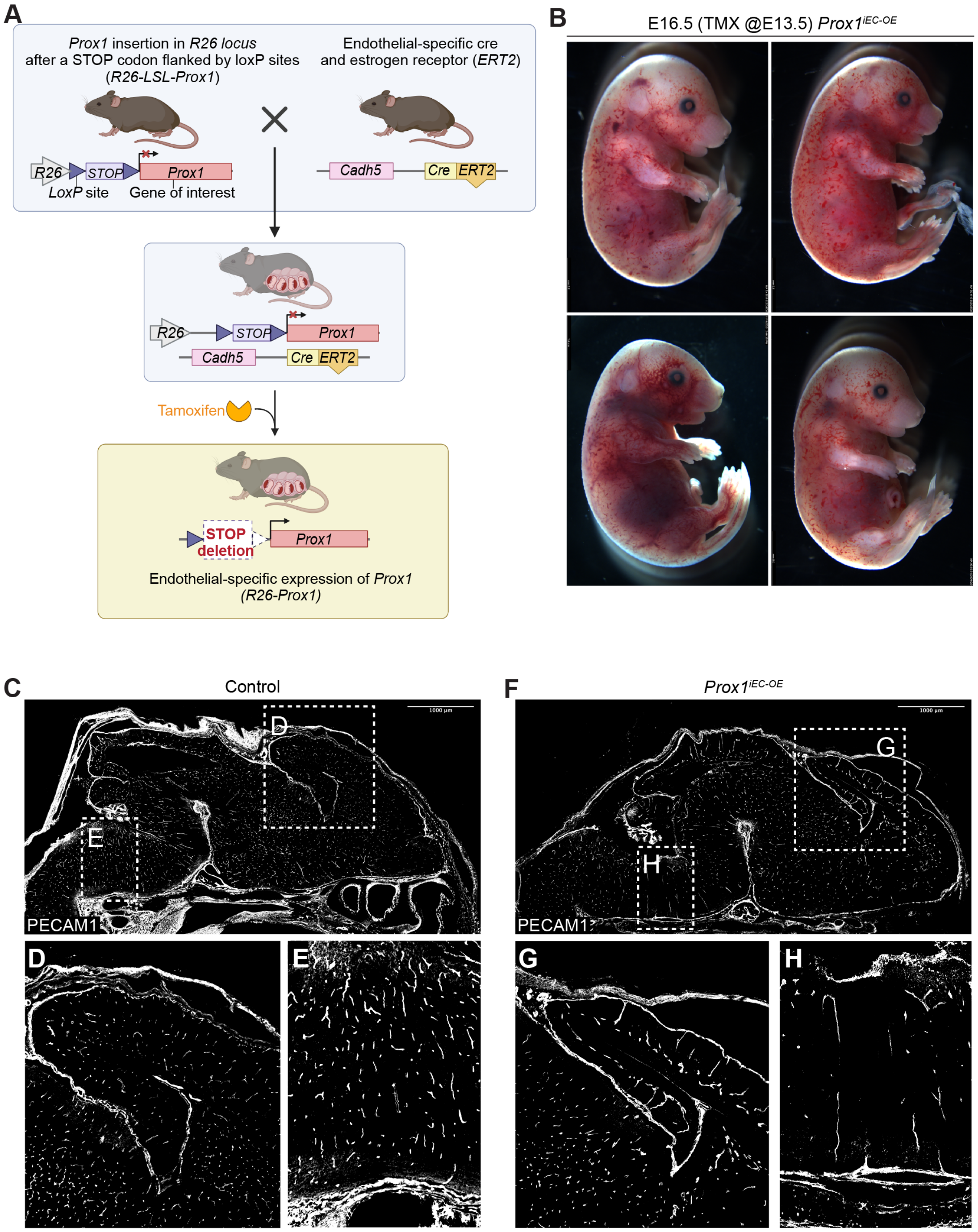

Supplemental Figure 4: Characterization of *Prox1$^{iEC\text{-}OE}$* embryos.
(**A**) Schematic diagram depicting the generation of *Prox1$^{iEC\text{-}OE}$* mice with EC-specific *Prox1* expression.
(**B**) Gross appearance of E16.5 *Prox1$^{iEC\text{-}OE}$* mutants induced at E13.5. Mutants exhibit edema, hemorrhage, and blood-filled lymphatics with variable severity.
(**C-H**) Sagittal brain sections from E16.5 controls (**C-E**) and mutants (**F-H**) stained for PECAM1 (grey). Boxed regions in (**C**, **F**) are magnified in (**D-E**, **G-H**). Scale bars: 1000 μm (**C**, **F**).

Supplemental Figure 5

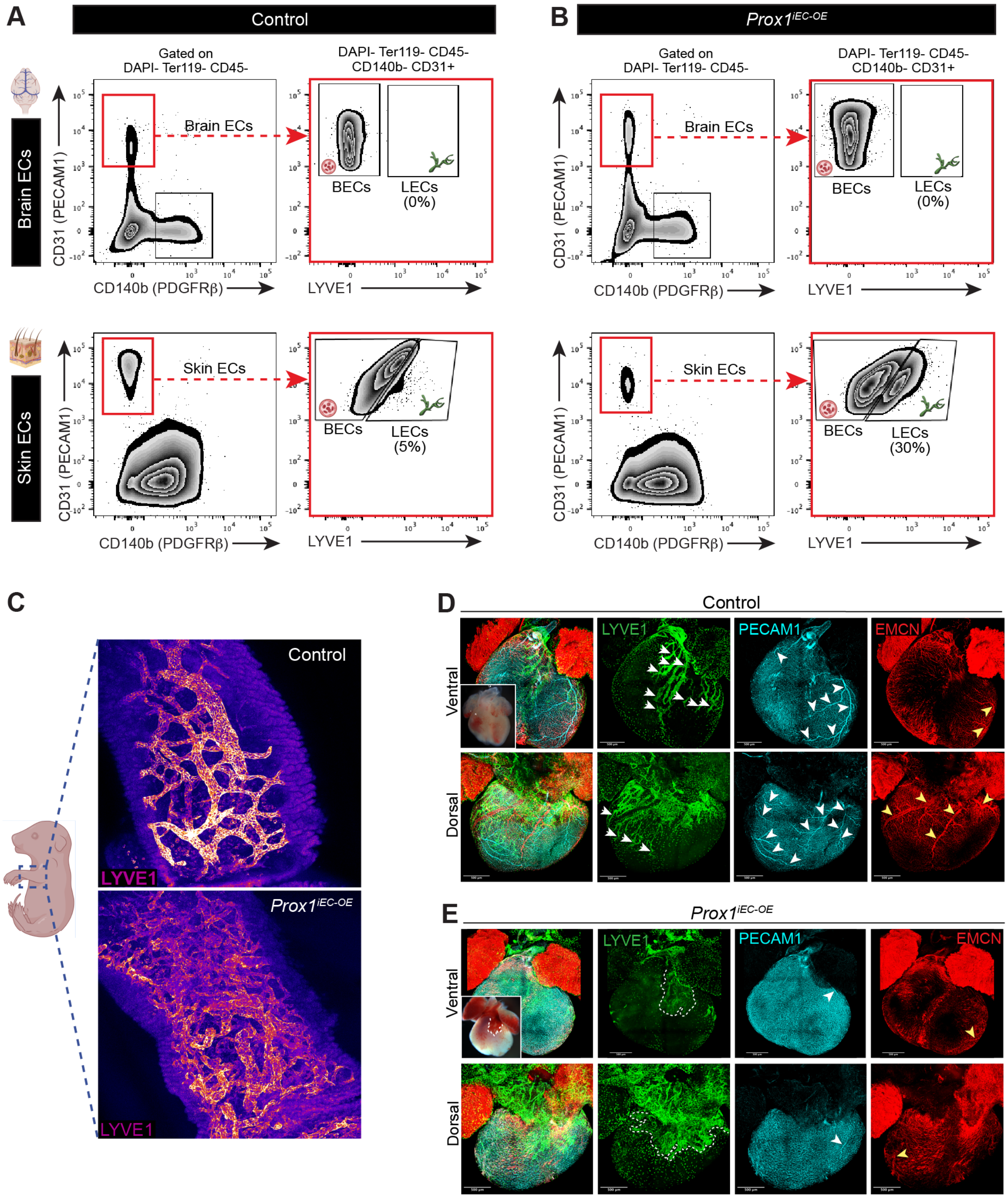

Supplemental Figure 5: Endothelial *Prox1* induces aberrant lymphatic vasculature in non-CNS tissues.

(**A-B**) Flow cytometry analysis of BECs (DAPI$^-$/Ter119$^-$/CD45$^-$/CD140b$^-$/CD31$^+$/LYVE1$^-$) and LECs (DAPI$^-$/Ter119$^-$/CD45$^-$/CD140b$^-$/CD31$^+$/LYVE1$^+$) from brain and skin of E16.5 *Prox1$^{iEC\text{-}OE}$* mutant and their control littermate embryos. *Prox1* overexpression increases LYVE1+ LECs in skin but not brain.

(**C**) Whole-mount immunostaining of limb skin from showing LYVE1+ lymphatics (fire LUT).

(**D-E**) Whole-mount immunostaining of heart ventricles from E16.5 controls (**D**) and *Prox1$^{iEC\text{-}OE}$* mutants (**E**) for LYVE1 (green), PECAM1 (cyan) and EMCN (red). Insets show blood-filled cardiac lymphatics in mutants. Arrows indicate LYVE1$^+$ lymphatics in controls; dashed outlines highlight aberrant LYVE1$^+$ vessels in mutants. Arrowheads (PECAM1) mark large coronary arteries; yellow arrowheads (EMCN) mark coronary veins. Scale bars: 500 µm.

Supplemental Figure 6

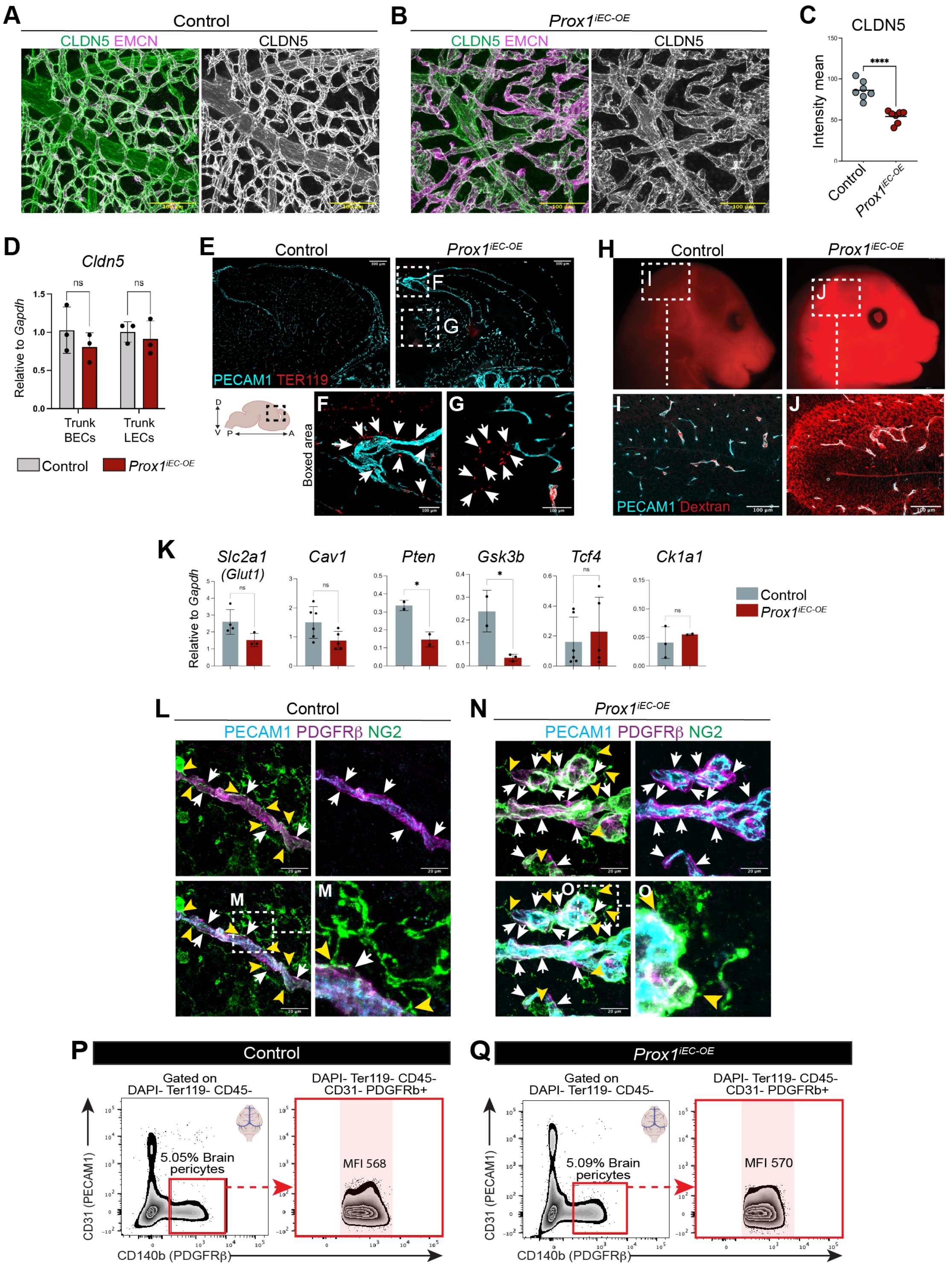

Supplemental Figure 6: Blood-brain barrier disruption in E16.5 *Prox1$^{iEC\text{-}OE}$* embryos.

(**A-C**) Whole-mount staining of control (**A**) and *Prox1$^{iEC\text{-}OE}$* mutant brains (**B**) for CLDN5 (grey/green) and EMCN (magenta). (**C**) Quantification of CLDN5 intensity mean (n=7-8 fields). ****p<0.0001, unpaired t-test. (**D**) qRT-PCR of *Cldn5* in FACS-isolated trunk BECs (PECAM1$^+$/LYVE1$^-$) and LECs (PECAM1$^+$/LYVE1$^+$) from E16.5 *Prox1$^{iEC\text{-}OE}$* mutants and their control littermates. n=3 independent experiments; ns, not significant. (**E-G**) Brain sections stained for PECAM1 (cyan) and TER119 (red). The boxed regions in (**E**, *Prox1$^{iEC\text{-}OE}$*) are magnified in (**F-G**). Arrows in (**F-G**) indicate extravascular TER119$^+$ blood cells in mutants.

(**H-J**) A tracer leakage assay of E16.5 *Prox1$^{iEC\text{-}OE}$* mutant and their control littermate brains with 3kDa dextran Texas-Red. The upper panels indicate gross appearance of E16.5 *Prox1$^{iEC\text{-}OE}$* mutant and their control littermate heads. The boxed regions in the upper panels largely correspond to the immunostaining images labeled with PECAM1 (cyan), displayed in the lower panels (**I-J**).

(**K**) Relative mRNA levels of BBB-related genes (*Slc2a1/Glut1*, *Cav1*, *Pten)* and *ß-catenin* target genes (*Gsk3b, Tcf4, Ck1a1)* in FACS- isolated brain ECs from E16.5 *Prox1$^{iEC\text{-}OE}$* mutant and control brains. n=2-6 biological samples from individual experiments. Mean ±SEM, unpaired t-test. Ns, not significant, *p<0.005.

(**L-O**) Section immunostaining of E16.5 *Prox1$^{iEC\text{-}OE}$* mutant and their control littermate brains with PECAM1 (cyan), PDGFRß (magenta), and NG2 (green). The boxed regions in the lower left panels of control (**L**) and mutant (**N**) are magnified in (**M** and **O**), respectively. Arrows mark PDGFRß$^+$/NG2$^+$ pericyte coverage of PECAM1$^+$ capillaries; yellow arrowheads indicate NG2$^+$/PDGFRß$^-$ oligodendrocytes associating with capillaries. (**P-Q**) Flow cytometry analysis of brain pericytes (DAPI$^-$/Ter119$^-$/CD45$^-$/CD31$^-$/CD140b$^+$) showing unchanged PDGFRß (MFI) between control and mutant embryos. n=3, Scale bars: 300 µm (**E**), 100 µm (**A-B**, **F-G**, **I-J**) ,20 µm (**L-N**).

Supplemental Figure 7

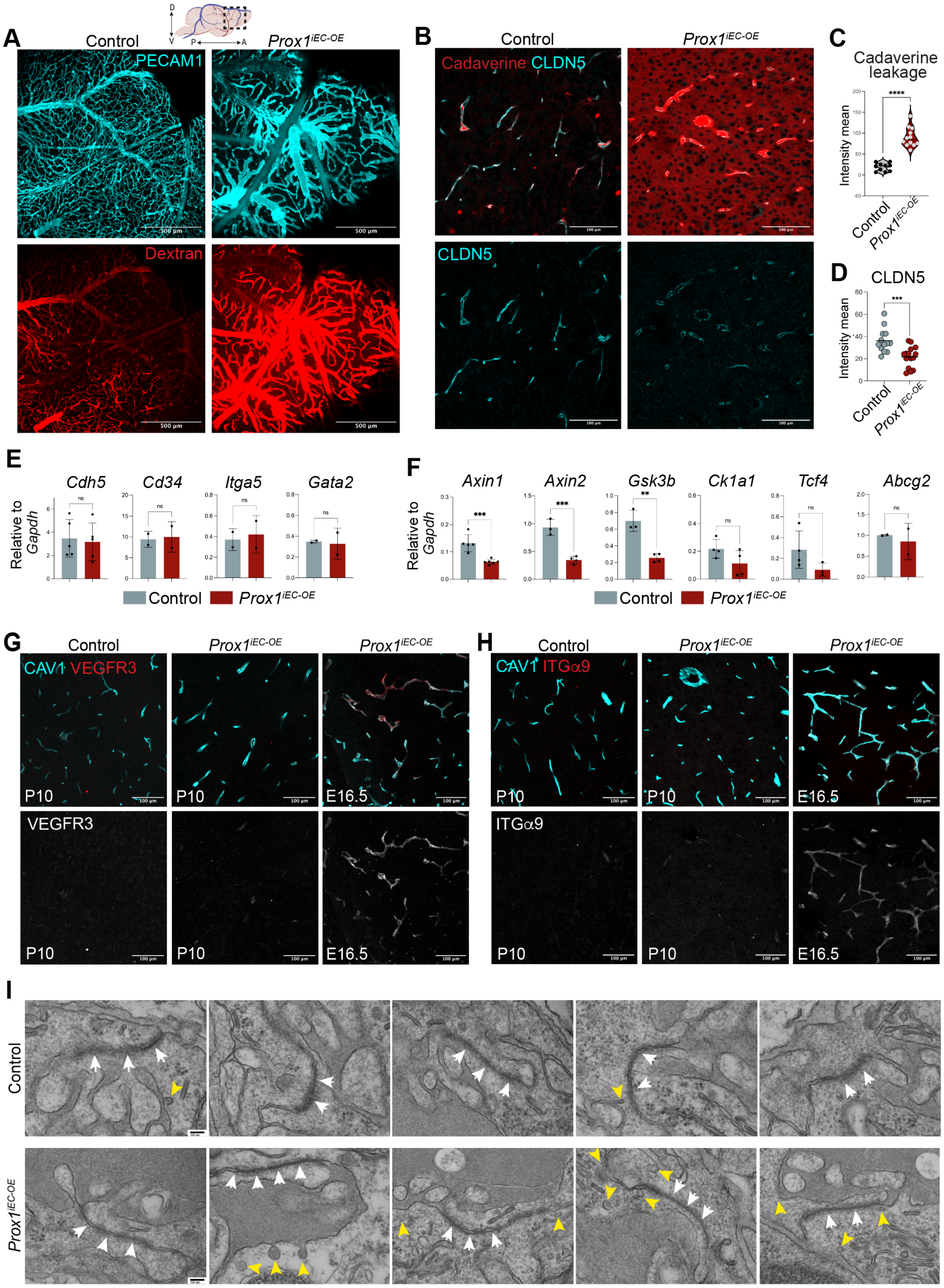

Supplemental Figure 7: Postnatal induction of *Prox1* disrupts the mature BBB without inducing hybrid blood-lymphatic phenotype.
(**A**) A surface view of whole-mount immunostaining of P10 $Prox1^{iEC\text{-}OE}$ mutant and their control littermate brain with 3kDa dextran (red) and PECAM1 (cyan).
(**B**) Section immunostaining of P10 $Prox1^{iEC\text{-}OE}$ mutant and their control littermate brain with 1kDa cadaverine tracer (red) and CLDN5 (cyan).
(**C-D**) Quantifications of cadaverine leakage (**C**) and CLDN5 intensity (**D**) in $Prox1^{iEC\text{-}OE}$ mutant and their control littermate brains. Dots represent random fields from ≥3 brains per group. ***p<0.0005, ****p<0.0001, unpaired t-test.
(**E-F**) Relative mRNA levels of BEC markers (*Cadh5*, *Cd34*, *Itga5*, *Gata2)* (**E**) and *ß-catenin* target genes (*Axin1, Axin2, Gsk3b, Ck1a1, Tcf4, Abcg2)* (**F**) in FACS-isolated brain ECs from P10 $Prox1^{iEC\text{-}OE}$ mutant and their control littermate brains (n=2-4 samples per group, 4 individual FACS experiments). Mean ±SEM, unpaired t-test. ns, not significant, **p<0.001, ***p<0.0005.
(**G-H**) Brain section stained with CAV1 (cyan) as a vascular marker, together with VEGFR3 (red and grey) in (**G**) or ITGα9 (red and grey) in (**H**).
(**I**) Representative TEM images showing structural differences in the tight junctions between ECs between control (top panels) and $Prox1^{iEC\text{-}OE}$ mutant (lower panels) brain vasculature (white arrows). Yellow arrowheads show increased number of vesicles in $Prox1^{iEC\text{-}OE}$ mutant brain ECs compared to controls. Scale bars: 500 µm (**A**), 100 µm (**B, G, H**), 100 nm (**I**).

Supplemental Figure 8

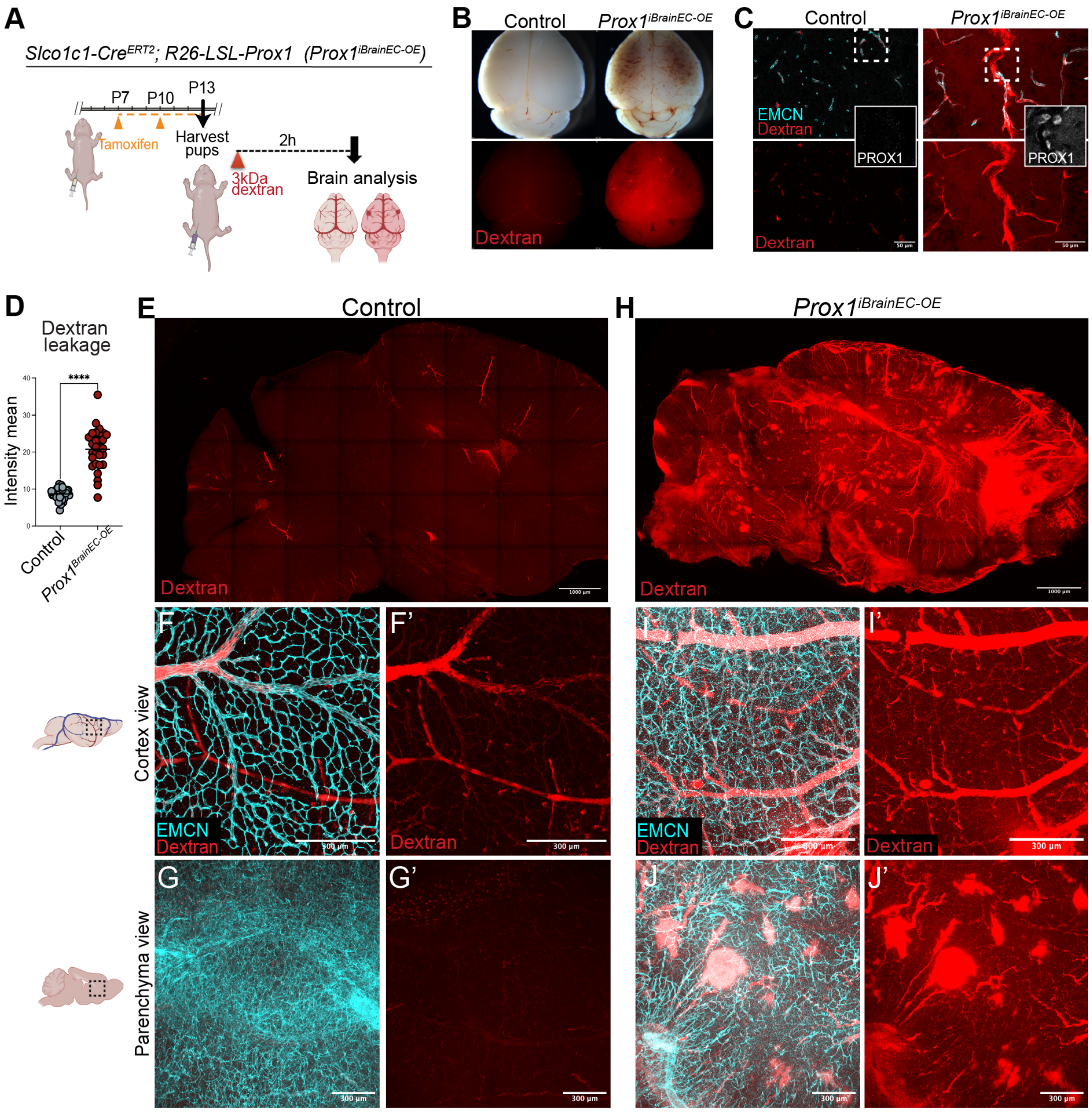

Supplemental Figure 8: Brain endothelial-specific *Prox1* overexpression disrupts BBB integrity.
(**A**) Schematic of the experimental design. *Prox1* overexpression was induced in brain ECs of *Slco1c1-Cre^{ERT2}; R26-LSL-Prox1* (*Prox1$^{BrainEC-OE}$*) mice by tamoxifen at P7 and P10; brains were collected at P13 after 3kDa dextran injection.
(**B**) Brightfield and fluorescent whole-mount images showing vascular abnormalities, hemorrhages, and dextran leakage in mutants.
(**C**) Brain sections stained for EMCN (cyan) and PROX1 (grey) confirms *Prox1* induction in mutants and associated dextran leakage (red).
(**D**) Quantification of dextran fluorescence intensity in brain parenchyma (n=29-30 images from 2 brains/group). Data are mean ± SEM. ****p<0.0001, unpaired t-test.
(**E–J**) Whole-mount sagittal images of cleared brains stained for EMCN (cyan) and imaged for 3kDa dextran leakage (red). Cortical vessels show extensive leakage in mutants (**I-I’**) vs controls (**F-F’**). Deep parenchymal vessels similarly exhibit widespread leakage and vessel disorganization in mutants (**J-J’**) vs controls (**G-G’**). Scale bars: 1000 µm (**E, H**), 300 µm (**F-G’**, I-J’), 50 µm (**C**).

Supplemental Figure 9

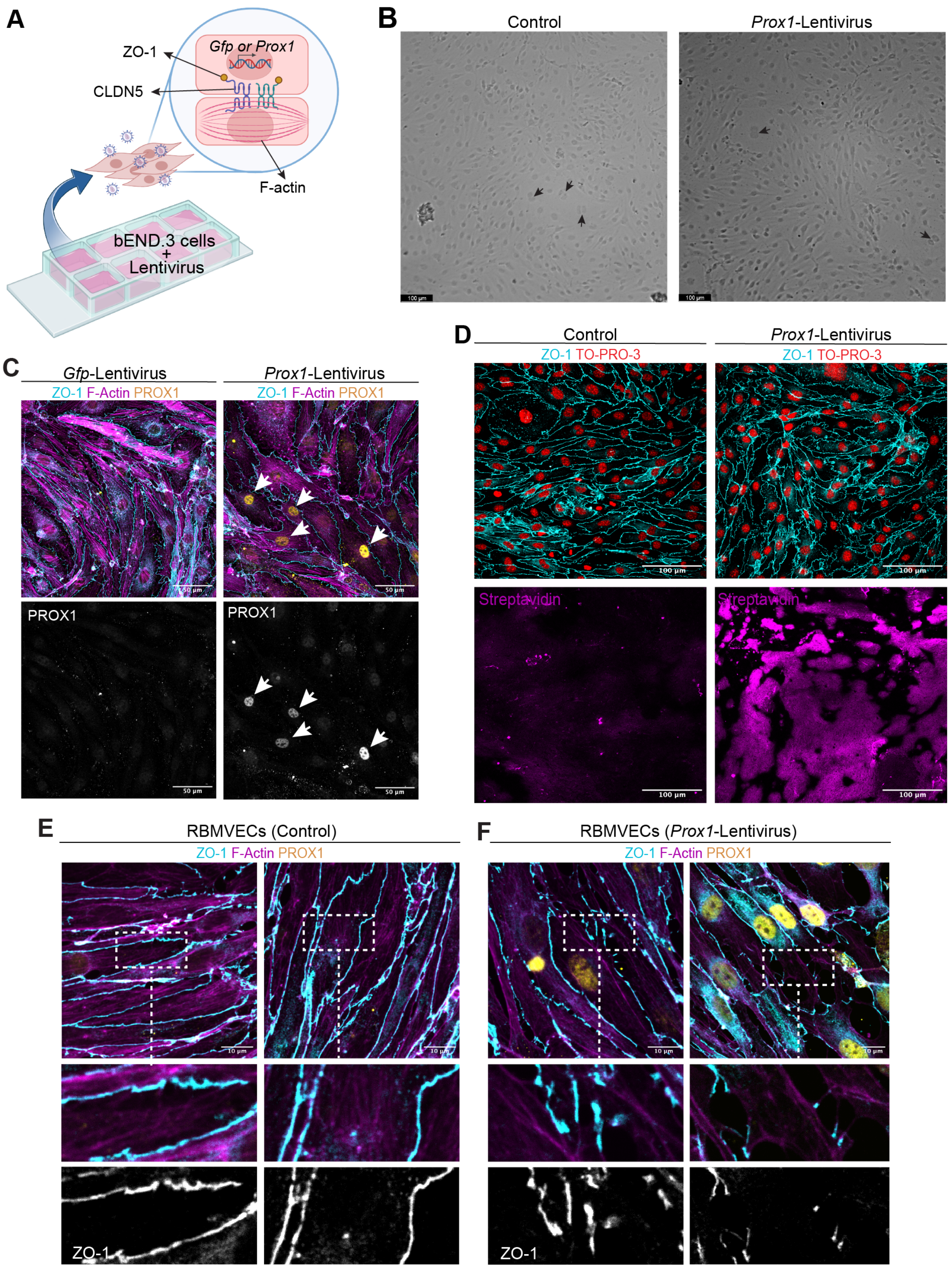

Supplemental Figure 9: PROX1 disrupts endothelial junction integrity in cultured brain ECs.
(**A**) Schematic diagram depicting the preparation of bEnd.3 cells expressing *Gfp or Prox1*. bEnd.3 cells are cultured in 8-12 well Ibidi chamber slides and transduced with empty lentivirus expressing *Gfp* (*Gfp*-Lentivirus) or *Prox1* gene (*Prox1*-Lentivirus) to study the effect of *Prox1* expression in the tight junctions. (**B**) Representative brightfield images of bEnd.3 cells infected with *Gfp* (control) or *Prox1*-lentivirus showing similar confluency and cell density between conditions (arrows indicate dividing cells).
(**C**) Immunostaining of bEnd.3 cells expressing *Gfp or Prox1* with PROX1 (yellow and grey), ZO-1 (cyan) and F-Actin (magenta). Arrows in bEnd.3 cells expressing *Prox1* indicate PROX1+ cells. bEnd.3 cells expressing *Gfp* do not express PROX1 antibody.
(**D**) Biotin matrix assay showing increased permeability in *Prox1*-expressing cells, detected by elevated streptavidin signal (magenta). Tight junctions are labeled with ZO-1 marker (cyan) and nuclear counterstain with TO-PRO-3 (red).
(**E-F**) Representative images of cultured rat brain microvascular ECs (RBMVEC) stained for ZO-1 (cyan/ grey), F-actin (magenta) and PROX1 (yellow). Insets show magnified regions. *Prox1* expression induces discontinuous ZO-1 junctions compared to controls. Scale bars: 100 µm (**B, D**), 50 µm (**C**),10 µm (**E-F**).

Supplemental Figure 10

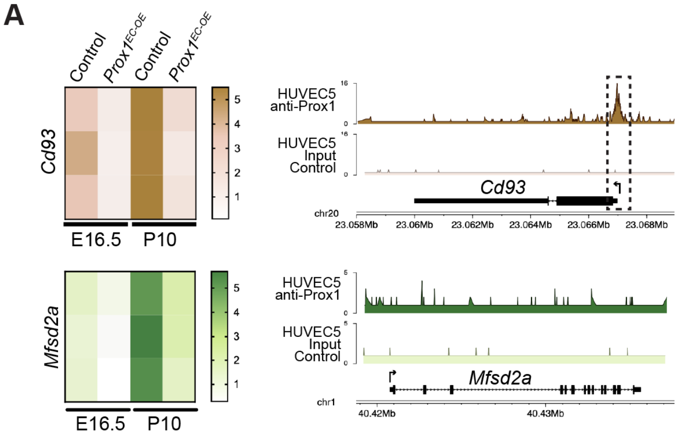

Supplemental Figure 10: PROX1 represses *Cd93* but not *Mfsd2a* expression.
(**A**) Heatmaps of relative mRNA expression of *Cd93* (top) and *Mfsd2a* (bottom) in brain ECs isolated from control and *Prox1*-overexpressing mice at E16.5 and P10, measured by qRT-PCR (left panel). A published whole-genome ChIP-seq study using anti-PROX1 antibody in human umbilical vein ECs (HUVECs) expressing *Prox1* (68) reveals the presence of PROX1-binding sites at the promoter of *Cd93* but not *Mfsd2a* (right panel). This data is available online through the Gene Expression Omnibus (GEO) under reference number GSE71230.

Supplemental Table 1: List of antibodies

REAGENT	SOURCE	IDENTIFIER
**Primary Antibodies for Immunostaining**		
Armenian Hamster anti-Pecam-1(clone 2H8)	Millipore	MAB1398Z
Rabbit polyclonal anti-Prox1	EMS Millipore	Cat#AB5475
Goat polyclonal anti-human Prox1	R&D	Cat#AF2727
Rabbit polyclonal anti-Lyve1	Abcam	Cat#ab14917
Rat monoclonal anti-Endomucin	Santa Cruz Biotech.	Cat#sc-65495
Mouse monoclonal anti-Claudin 5 (Clone 4C3C2), Alexa Fluor™ 488	Invitrogen	Cat#352588
Rabbit polyclonal anti-Claudin 5	ThermoFisher	Cat#34-1600
Rabbit polyclonal anti-NG2	Millipore-Sigma	Cat#AB5320
Rabbit polyclonal anti-ERG	Abcam	Cat#ab92513
Rat monoclonal anti-PLVAP (clone MECA-32)	BD Pharmigen	Cat#550563
Goat polyclonal anti-ITGa9	R&D	Cat#AF3827
Goat polyclonal anti-VEGFR3	R&D	Cat#AF743
Rabbit polyclonal anti-ZO-1	Proteintech	Cat#21773-1-AP
Rat monoclonal anti-PDGFRb (clone APB5)	eBioscience	Cat#14-1402
Goat polyclonal anti-GFP	abcam	Cat#Ab6673
Alexa Fluor 568 Phalloidin	Invitrogen	Cat#A12380
Rabbit polyclonal anti-CAV-1 (clone D46G3)	Cell signaling	Cat#3267T
**Secondary Antibodies for Immunostaining**		
Goat polyclonal anti-Rabbit, AlexaFluor® 488	Jackson Immunoresearch	Cat#111-547-003
Goat polyclonal anti-Rabbit, AlexaFluor® 594	Jackson Immunoresearch	Cat#111-586-047
Goat polyclonal anti-Rabbit, AlexaFluor® 647	Jackson Immunoresearch	Cat#111-605-144
Goat polyclonal anti-Rat, AlexaFluor® 488	Jackson Immunoresearch	Cat#112-545-003
Goat polyclonal anti-Rat, AlexaFluor® 594	Jackson Immunoresearch	Cat#112-585-167
Goat polyclonal anti-Rat, AlexaFluor® 647	Jackson Immunoresearch	Cat#112-605-003
Goat polyclonal anti-Armenian Hamster, AlexaFluor® 488	Jackson Immunoresearch	Cat#127-545-099
Goat polyclonal anti-Armenian Hamster, Cy3	Jackson Immunoresearch	Cat#127-165-160
Goat polyclonal anti- Armenian Hamster, AlexaFluor® 647	Jackson Immunoresearch	Cat#127-605-160
Donkey polyclonal anti-Rat, AlexaFluor® 488	Jackson Immunoresearch	Cat#712-545-150
Donkey polyclonal anti-Rat, AlexaFluor® 594	Jackson Immunoresearch	Cat#712-585-153
Donkey polyclonal anti-Rat, AlexaFluor® 647	Jackson Immunoresearch	Cat#712-605-150
Donkey polyclonal anti-Rabbit, AlexaFluor® 488	Jackson Immunoresearch	Cat#711-547-003
Donkey polyclonal anti-Rabbit, AlexaFluor® 594	Jackson Immunoresearch	Cat#711-587-003
Donkey polyclonal anti-Rabbit, AlexaFluor® 647	Jackson Immunoresearch	Cat#711-605-152
**Flow Cytometry Antibodies**		
Rat monoclonal anti-NG2, AlexaFluor® 488	Millipore Sigma	Cat#AB5320A4
Rat monoclonal anti-CD45 (30-F11)	eBioscience	Cat#14-0451-82
Rat monoclonal anti-TER-119	eBioscience	Cat#19-5921-85
Rat monoclonal anti-CD140b (APB5), APC-conjugated	eBioscience	Cat#17-1402-82
Rat monoclonal anti-CD31 (390), PE-Cyanine7 conjugated	eBioscience	Cat#25-0311-82
Rat monoclonal anti-CD45, Brilliant Violet 785™	Biolegend	Cat#103149
Rat monoclonal anti-Ter119, Brilliant Violet 785™	Biolegend	Cat#116245
Rat monoclonal anti-LYVE1 (ALY7), AlexaFluor® 488	eBioscience	Cat#53-0443-82
Rat monoclonal anti-LYVE1, PE-conjugated	R&D	Cat#FAB2125P

Supplemental Table 2: List of primers for qRT- PCR

Gene	**Primer Forward (5'-3')**	**Primer Reverse (5'-3')**
Gadph	ctgcaccaccaactgcttag	tctcatcatacttggcaggt
Prox1	agaagggttgacattggagtga	tgcgtgttgcaccacagaata
Claudin-5	acatgcagtgcaaggtgtat	ggtaacaaagagtgccacca
Plvap	gctggtactacctgcgctatt	cctgtgaggcagatagtcca
Sox17	acgctagctcagcggtctactatt	agggatttccttagcgcttccagg
Ctnnb1	gttcgccttcattatggactgcc	atagcaccctgttcccgcaaag
Lef1	actgtcaggcgacacttccatg	actgtcaggcgacacttccatg
Glut1	gcttctccaactggacctcaaac	acgaggagcaccgtgaagatga
Axin1	acgaggagcaccgtgaagatga	gcccattgacttggatactctcc
Axin2	atggagtccctccttaccgcat	gttccacaggcgtcatctcctt
Fzd4	actttcacgccgctcatccagt	tctcaggactggttcacagcgt
Fgfbp1	acggagccaaacagggtcaaag	ggtcttcctctggttgagcaca
Apcdd1	cggtgtgctctcatctaaggtc	cccactgaagacattgaggagg
Itgα*5*	cttctccgtggagttttaccg	cttctccgtggagttttacc
Cd34	ggtagctctctgcctgatgag	tggtaggaactgatggggatatt
Gata2	caccccgccgtattgaatg	cctgcgagtcgagatggttg
Gsk3b	ctttggaagtgcaaagcag	ccaactgatccacaccac
Mfsd2a	ctcctggccatcatgctctc	ggccaccaagatgagaaa
Zo-1	gccgctaagagcacagcaa	tccccactctgaaaatgagga
Ve-cadherin	aaccatgacaacaccgcca	cgttgtctgagatgagcacg
Cav1	gcgaccccaagcatctcaa	atgccgtcgaaactgtgtgt
Cd93	gatggctctttctactgctcctg	ccacacctgaaggaaccatctg
Pten	tgagttccctcagccattgcct	gaggtttcctctggtcctggta
Abcg2	cagttctcagcagctcttcgac	tcctccagagatgccacggata
Tcf4	cactttccctagctccttcttc	gttcgtgtggtcaggagaatag
Ck1a1	tagctgaccagatgatcag	gtatcgggcagtgccagtg
